# Entropy-Based Uncertainty Management and Decision Support Under Strategic Agent Interactions in Institutional Survey Systems

**DOI:** 10.3390/e28080839

**Published:** 2026-07-27

**Authors:** Cemil Gündüz, Üzeyir Fidan, Ali Erbey

**Affiliations:** 1 Department of Computer Programming, Distance Education Vocational School, Usak University, Uşak 64200, Türkiye; uzeyir.fidan@usak.edu.tr; 2 Department of Management Information Systems, Faculty of Economics and Administrative Sciences, Tarsus University, Mersin 33400, Türkiye; alierbey@tarsus.edu.tr

**Keywords:** Shannon entropy, Kullback–Leibler divergence, strategic data manipulation, decision support systems, game theory, decision-making under uncertainty

## Abstract

Institutional governance systems increasingly rely on stakeholder surveys in strategic decision-making. Yet survey participants can act as strategic agents who shape organizational outcomes in their favor. By transforming the information content of response distributions, such behavior can systematically distort institutional decisions made under uncertainty. This study proposes a dynamic framework that detects strategic data manipulation in institutional surveys using Shannon entropy and Kullback–Leibler divergence, and converts this detection into decision support. The framework operates in three stages. First, it constructs a robust reference entropy profile from historical data. Second, it processes incoming survey responses as a sequential stream and compares them against this profile. Third, it detects manipulation at both the population and individual levels through a multi-layered anomaly scoring system. The reference profile was built from anonymized real survey data spanning 2021–2025, comprising 1233 participants and 19,728 clean observations. The framework was validated through 600 Monte Carlo scenarios derived from this profile, covering four manipulation types and five intensity levels, and was benchmarked against the Z-score and Isolation Forest methods. The findings are threefold. Straight-lining detection identifies individual suppression and inflation manipulations with perfect accuracy. KL divergence monitoring flags coordinated coalition entries before data collection is complete. Hierarchical clustering recovers a coordinated suppression group that individual scoring fails to isolate, with 76.5% cluster purity and ~59% recall. Policy impact analysis further shows that manipulation distorts dimensions in opposite directions: the gap between raw and verified means is positive in the dimension targeted by coordinated suppression but clearly negative in the dimension targeted by coordinated inflation. This bidirectional distortion shows why dimension-selective detection is necessary, as a single uniform correction cannot resolve it. The study contributes to the literature in two areas, integrating decision-making under uncertainty with strategic agent models, and survey integrity research with information-theoretic metrics.

## 1. Introduction

Modern institutions increasingly base their decisions on data. Stakeholder surveys play a role as a fundamental decision input in the resource allocation, strategic planning, and policy development processes of universities, public institutions, and large organizations [1,2]. These surveys function as a mechanism that transforms distributed stakeholder knowledge into institutional intelligence. The central position of the stakeholder concept in corporate governance is grounded in the stakeholder approach, which dictates that organizations must systematically gather the opinions of not only internal actors but all groups whose interests are affected by institutional decisions [3].

However, this process contains a structural difficulty. Survey participants are not passive sources of information. Academic units, administrative groups, and other institutional actors perceive survey outputs as signals that directly affect their own interests. This perception can lead stakeholders to respond in a manner that manipulates the collective outcome in their favor rather than reporting their true preferences. The behavior in question poses a limited threat when it remains individual. However, when it transforms into a coordinated group behavior, those making the strategic decisions of the institution are forced to rely on distorted data.

This difficulty is not an abstract concern; it was concretely observed in the institutional dataset on which this study is based. We examined five years of academic staff satisfaction survey data from a public university, covering 2021 to 2025. Providing identical responses to all questions is known as straight-lining. No participant exhibited this pattern in any year from 2021 to 2024. In 2025, however, participants exhibiting this pattern emerged for the first time. Straight-lining behavior is merely the crudest and most visible form of the strategic response repertoire. The initial emergence of this visible signal from a stable baseline constitutes a concrete warning that more subtle and coordinated forms of strategic behavior might also exist, and these cannot be captured by a simple screening. In an institutional context where survey results directly feed resource allocation, program evaluation, and strategic planning decisions, this observation directly demonstrates the need for a systematic monitoring capacity. The decision-support framework developed in this study was born precisely out of this need.

The study examines strategic signal manipulation in institutional survey systems at the intersection of decision theory and information theory. The primary objective of the research is to detect manipulation-induced data uncertainty through Shannon entropy and Kullback–Leibler divergence, and to develop a framework that transforms this detection into institutional decision support.

The literature on decision-making under uncertainty generally treats data uncertainty as an exogenous and constant characteristic. Noise, missing data, and measurement errors are the standard objects of this approach. However, a different type of uncertainty comes into play in institutional survey environments. This uncertainty is not exogenous but endogenous, originating from within the data collection process and the conscious decisions of strategic actors. Strategic manipulation transforms the information-theoretic structure of the response distribution. While coordinated suppression behavior artificially erodes the natural diversity of opinions, coordinated inflation behavior creates a similar distortion from the opposite direction. In both cases, Shannon entropy deviates from the level expected had the manipulation not occurred. This deviation can be quantified through the distance to the reference distribution estimated from historical data, representing the KL divergence. In other words, strategic manipulation leaves a unique trace in the data space, and entropy is the statistical indicator of this trace. This relationship forms the theoretical backbone of the framework.

Three distinct fields of literature partially address this problem, yet none offers a sufficient response on its own. Survey quality research has extensively documented response errors; however, these studies position the participant as a passive object of measurement and do not structurally include the assumption of intentional and coordinated manipulation [4,5]. Data poisoning research in machine learning has mathematically demonstrated how model behavior is deflected by corrupted data, but these studies model exogenous attacks and do not cover human agent manipulation arising endogenously from institutional incentive structures [6,7]. Entropy-based decision-making research has successfully integrated information-theoretic metrics into multi-criteria decision frameworks, yet in these studies, data uncertainty is treated as a constant and passive input, and the roles of strategic agents actively distorting the data are not modeled [8,9]. A clear gap exists at the intersection of these three fields, as data uncertainty arising from strategic interactions in a multi-agent institutional environment has not been systematically addressed with entropy-based methods. This study aims to fill the gap in question and seeks answers to the following four research questions.

RQ1: Can strategic manipulation in institutional survey systems be statistically detected through temporal deviations in entropy and KL divergence?

RQ2: What are the statistical operating characteristics of the proposed framework across different manipulation types and intensity levels?

RQ3: Do individual strategic response misreporting and coordinated coalition behavior leave distinguishable signatures on entropy dynamics?

RQ4: How are detection outputs transformed into institutional decision support, and what is the magnitude of undetected manipulation reflected as decision error?

Through its answers to the research questions, this study provides four primary original contributions to the literature. First, the institutional survey system is formalized as a multi-agent decision environment, and the effects of strategic manipulation on entropy are mathematically characterized. Second, a three-stage dynamic entropy-monitoring framework that does not require labeled training data is developed, offering a methodological contribution that enables manipulation detection at both the population and individual levels. Third, comprehensive Monte Carlo experiments covering four manipulation types and five intensity levels are conducted on a reference profile derived from actual university survey data, and the statistical operating characteristics of the framework are determined comparatively against standard anomaly-detection methods such as the Z-score and Isolation Forest. Fourth, an institutional decision-support architecture incorporating a confidence score system, policy impact analysis, and early warning capacity is designed, documenting the quantitative impact of undetected manipulation on decision errors.

The remainder of the study is organized as follows. Section 2 presents the theoretical framework and the related literature. Section 3 details the three-stage dynamic entropy-monitoring framework. Section 4 addresses the construction of the reference profile over five years of actual institutional data. Section 5 presents the decision-support architecture and validates the framework on a controlled test set. Section 6 discusses the findings, and Section 7 summarizes the conclusions.

## 2. Theoretical Framework and Related Work

### 2.1. Decision-Making Under Uncertainty and Survey-Based Decision-Support Systems

Institutional decision-making is designed as a rational process based on complete and reliable information under ideal conditions. However, this ideal is rarely realized in practice. Simon [10] established that the cognitive capacities of decision-makers and the information available to them are always limited. March and Simon [11] extended this bounded rationality-based understanding to the organizational level, demonstrating that institutional decisions are made under cognitive and informational constraints. This limitation renders making institutional decisions under uncertainty inevitable.

Types of uncertainty require a critical distinction in this context. Epistemic uncertainty stems from a lack of knowledge and can in principle be reduced by collecting more data. Aleatory uncertainty refers to the irreducible randomness inherent in the nature of events [12,13]. Institutional decision-support systems are designed to manage these two types of uncertainty. Stakeholder surveys are among the most widely used data sources for these systems. Large public institutions, particularly universities, administer periodic surveys to evaluate service quality, justify resource allocation and determine strategic priorities [1,2]. These surveys gather distributed stakeholder knowledge to construct a collective representation at the institutional level, and decision-makers utilize this representation as a reliable input.

The existing decision-support literature treats survey data quality largely as a technical measurement issue. Sample size scale validity and reliability coefficients are the primary focus areas of this approach [14,15]. Methodological discussions regarding the use and interpretation of Likert-type scales also constitute a significant part of this literature [16]. Survey response behavior research has documented response strategies developed by participants under cognitive load, such as superficial processing or random responding [17]. While these studies are valuable, they are built upon a common assumption, which is the premise that participants structure their responses honestly and independently. This premise is a critical prerequisite for institutional evaluation processes and can be systematically violated in institutional survey environments. Survey results often directly influence resource allocation, program evaluation, and performance decisions. In such settings, the response behavior of the participant moves beyond pure information transmission. The participant ceases to be a passive measurement object and instead becomes a strategic actor driven to shape the outcome. This transformation creates a new type of uncertainty that existing decision-support models ignore.

At this point, data integrity research within the machine learning literature offers a conceptual bridge. Data poisoning studies have mathematically demonstrated how corruptions intentionally introduced into training data deflect model behavior [6,7]. The influence function approach has quantified the disproportionate impacts of individual observations on model outputs [18]. This literature is close to the present study in terms of formally characterizing the impact of corrupted data on decision outputs. However, the studies in question model exogenous and algorithmic attacks and do not encompass human agent and coordinated manipulation arising endogenously from institutional incentive structures. This study extends the data integrity perspective to the institutional survey context.

This study formalizes the institutional survey system as a multi-agent decision environment. The system is defined as S=(I, Q, A, Ω, U, R) where

$I=\left\{1,\;\ldots ,\;n\right\}$ denotes the set of participants or strategic agents.

$Q=\{q_{1},\;\ldots ,\;q_{m}\}$ represents the set of questions.

A={1, …, k } defines the Likert scale response space.

Ω represents the true and unobservable preference distribution.

For each agent i∈I and question q∈Q, $\omega_{iq}\in \Omega$ denotes the true, unobservable preference of agent i regarding question q, and f:Ω→A denotes the sincere reporting function that maps this preference to the Likert category best representing it. The equality $r_{iq}=f\left(\omega_{iq}\right)$ therefore states that the reported answer coincides with the sincere image of the true preference.

$U_{i}(\cdot )$ is the utility function of agent i and encompasses the institutional gains obtained by the agent from the survey output.

R:I×Q→A specifies the response strategy of each agent for each question.

Two fundamental definitions are presented based on this formalization.

**Definition** **1.** *Honest Response: Agent *i *provides an honest response if they report the answer reflecting their true preference for question *q*. In this case the relationship *$r_{iq}=f(\omega_{iq})$ *holds.*

**Definition** **2.** *Strategic Response: Agent *i *provides a strategic response if they report *$r_{iq}\neq \;f(\omega_{iq})$ *to influence the collective outcome in a manner that increases their own utility.*

This distinction forms the basis of the entire subsequent theoretical structure. The mathematical consequences of manipulation are examined in detail in Section 2.3 and Appendix A.

### 2.2. Strategic Agent Interactions and a Game-Theoretic Perspective

The sustainability of honest information transmission in environments where multiple agents share a common information channel is one of the fundamental questions long examined by game theory. Crawford and Sobel [19] demonstrated that transmitted signals are systematically distorted when the interests of the sender and the receiver diverge. According to this “cheap talk” model, complete and honest information transmission is possible only when interest alignment is achieved, when the signal is costless. Subsequent studies expanded this framework and mapped in detail under which conditions cheap talk facilitates or hinders information transmission [20]. The issue of the robustness of collective decision mechanisms against strategic manipulation lies at the center of social choice theory. Arrow [21] showed that an ideal aggregation rule transforming individual preferences into a consistent collective preference cannot exist under certain reasonable conditions. Based on this impossibility result, Gibbard [22] and Satterthwaite [23] proved that no voting mechanism can be immune to strategic manipulation under certain conditions. Myerson [24] formalized the incentive-compatibility condition and the revelation principle, establishing the basis for mechanisms that make honest reporting a dominant strategy. Together these theoretical frameworks reveal that, in every collective decision environment where a conflict of interest exists, strategic behavior is a structural, constitutive meaning rather than an accidental phenomenon. Information theory has more recently been turned towards strategic behavior itself: entropy has been used to quantify the complexity and predictability of a player’s mixed strategy, tying the unpredictability of strategic play directly to an information measure [25].

The literature on coalition formation and power distribution adds an additional dimension to this picture. Shapley and Shubik [26], along with Banzhaf [27], measured the disproportionate impacts of coalitions on the collective outcome in multi-actor decision systems through power indices. Coordinated group behavior carries an impact capacity far beyond individual strategic responses. The organizational behavior literature has extended these abstract predictions to the institutional context. Pfeffer and Salancik [28] documented that institutional groups strategically withhold or distort information under the pressure of resource dependence. Cyert and March [29] demonstrated that organizations do not consist of a single rational actor but rather coalitions with conflicting interests, and that these coalitions guide decision processes in line with their own agendas.

A significant portion of this literature remains at the theoretical level. A functional framework that detects the empirical traces of strategic behavior in survey systems and integrates this detection into decision support has not been developed. Game theory answers the question of why, while the question of how it is to be detected remains unanswered.

This study establishes a strategic agent typology specific to the institutional survey context and defines four primary manipulation types.

Type I Suppression: An agent or coalition systematically reports low scores for specific dimensions. The objective is to lower the evaluation of the relevant unit.

Type II Inflation: An agent or coalition systematically reports high scores. The objective is to present their own unit more favorably than would be justified under the circumstances.

Type III Noise Injection: An agent provides inconsistent and random responses. The objective is to reduce the information content of the survey signal.

Type IV Coordinated Coalition: Multiple agents exhibit Type I or Type II behavior in a coordinated manner, carrying a much larger output-deflection capacity than individual strategic behavior. Two sub-types are distinguished: Type IV-A (Coordinated Suppression), in which the coalition jointly reports low scores on a shared target dimension, and Type IV-B (Coordinated Inflation), in which the coalition jointly reports high scores on its target dimension. These sub-types are referred to as Type IV-A and Type IV-B throughout the analysis.

The impact of each manipulation type on entropy differentiates. Type I and Type II lower Shannon entropy, while Type III increases it and Type IV simultaneously disrupts both the entropy in the target dimension and the interdimensional correlation structure. These distinct signatures play a decisive role in the design of the detection mechanism.

The study also proposes a Stackelberg game model. In this model, the system designer is the leader and selects the mechanism determining the detection probability whereas the strategic agents act as followers determining the optimal manipulation intensity in response to the detection probability.

The central result of this model is the following deterrence proposition.

**Proposition** **1** (Deterrence)**.** *In the Stackelberg game between the system designer (leader) and the strategic agent or coalition (follower), let the follower choose a manipulation intensity* δ≥0 *maximizing the expected utility* $U\left(\delta \right)=\left(1-\pi \left(\delta \right)\right)\;G\left(\delta \right)-\pi \left(\delta \right)\;K$*, where* G *is the gain from undetected manipulation with* $G\left(0\right)=0$*,* $G^{\prime }\left(\delta \right)>0$*, and* $G^{\prime \prime }\left(\delta \right)\leq 0$*;* K>0 *is the penalty incurred upon detection; and* $\pi \left(\delta \right)\in \left[0,1\right]$ *is the detection probability, non-decreasing and differentiable in* δ *with* $\pi \left(0\right)=\pi_{0}$*. If the marginal deterrence dominates the marginal gain, *

$$\pi^{\prime }\left(\delta \right)\left(G\left(\delta \right)+K\right)\geq \left(1-\pi \left(\delta \right)\right)G^{\prime }\left(\delta \right)\;\;\mathrm{for}\;\mathrm{all}\;\delta \geq 0,$$

*and in particular, if* $\pi^{\prime }\left(0\right)\geq G^{\prime }\left(0\right)/K$*, then the unique best response is* $\delta^{*}=0$*: honest reporting is the dominant strategy and the equilibrium manipulation intensity collapses to zero.*

In other words, an effective detection mechanism functions as a deterrent, making responding honestly the dominant strategy. The proof is presented in Appendix A.1.

### 2.3. Theoretical Foundations of Entropy and KL Divergence Under Decision Uncertainty

Throughout this subsection, let $\left(\Omega ,F,P\right)$ denote a probability space, where Ω is the sample space, F is a σ-algebra of events on Ω, and P is a probability measure on F. Survey responses are modeled as a discrete random variable X:Ω→A defined on this space and taking values in the finite Likert response set A={1,…,k}. Every probability distribution considered below is the probability mass function induced by such a random variable; that is, $P=\{p_{1},\ldots ,p_{k}\}$ with $p_{a}=P\left(X=a\right)$, $p_{a}\geq 0$ for all a∈A, and $\sum_{a=1}^{k}p_{a}=1$. Shannon [30] defined entropy, which mathematically measures the uncertainty of the information source represented by X. For the distribution P, Shannon entropy is expressed through Equation (1).

(1) $$H\left(P\right)=-\sum_{i=1}^{k}p_{i}\cdot {log}_{2}\left(p_{i}\right)$$

This metric numerically demonstrates how uniform or how concentrated the distribution is. High entropy reflects high uncertainty, whereas low entropy reflects high predictability. Jaynes [31] developed the maximum entropy principle, stating that among the distributions consistent with current constraints, the one that assumes the least information, which means the one having the highest entropy, should be selected. This approach forms the information-theoretic basis for constructing a priori under uncertainty and is directly connected to Bayesian inference. The formal properties of entropy and related metrics are comprehensively addressed in the standard framework of information theory [32].

Kullback and Leibler [33] defined a divergence that measures the information distance between two probability distributions. Let P and Q be probability mass functions defined on the same finite space A, induced by A-valued random variables on $\left(\Omega ,F,P\right)$, and assume absolute continuity, written P≪Q; that is, $Q\left(x\right)=0$ implies $P\left(x\right)=0$ for every x∈A. Under the standard convention $0\cdot {log}_{2}\left(0/q\right)=0$ for q≥0, the Kullback–Leibler divergence is given by Equation (2):

(2) $$D_{KL}\left(P∥Q\right)=\sum_{x\in A}P\left(x\right){log}_{2}\;\left(\frac{P\left(x\right)}{Q\left(x\right)}\right)$$

By Gibbs’ inequality, $D_{KL}\left(P∥Q\right)\geq 0$, with equality if and only if P=Q; the absolute-continuity condition guarantees that the divergence is finite. This metric expresses the expected information loss resulting from utilizing distribution Q for coding instead of P. KL divergence lies at the theoretical foundation of model selection as well as distribution comparison and Bayesian updating [34].

Entropy and KL divergence have found wide application in the field of multi-criteria decision-making. Rough set theory, introduced by Pawlak [8], and three-way decision frameworks developed by Yao [9] model uncertainty through information-theoretic tools. Fuzzy set theory, proposed by Zadeh [35], and the Dempster–Shafer theory of evidence formulated by Dempster [36] stand out as approaches complementing this framework. Decision rule-based approaches discussed by Greco et al. [37] and the ordered weighted averaging operators presented by Yager and Kacprzyk [38] have advanced the integration of information-theoretic metrics into multi-criteria decision problems further. More recent contributions continue to operationalize entropy as an objective criterion-weighting mechanism within institutional decision-support systems, for example, by combining an extended entropy weighting method with multi-criteria aggregation to rank policy alternatives [39], or by hybridizing entropy-based information weights with robust dispersion measures to stabilize criterion weighting under data contamination [40]. This literature treats data uncertainty as an exogenous and constant system property, positioning data producers as passive sources. Therefore, existing entropy-based decision frameworks do not model the roles of strategic actors interfering with the data production process.

One of the most recent advancements regarding this limitation is in studies extending entropy-based metrics to multi-agent and federated decision environments, such as the work in [41]. The approach in question demonstrates the central role of entropy in combining information from distributed agents under uncertainty. Nevertheless, these studies also treat agents as noisy but well-intentioned information sources rather than strategic actors driven to corrupt the outcomes in their favor. When the source of uncertainty is strategic and located within the data collection process, existing frameworks remain inadequate.

This study equips Shannon entropy and KL divergence with a function specific to the institutional survey environment. Shannon entropy is utilized as a metric for response diversity within survey dimensions. The value $H(Q_{q})$ calculated for question q reflects the opinion distribution of participants in that dimension. When manipulation does not occur, this value is expected to remain within a specific band and any departure from this band constitutes a warning signal.

**Proposition** **2** (Manipulation-induced divergence)**.** *Fix an item* q *with reference distribution* $P_{ref}=\left(p_{1},\ldots ,p_{k}\right)$ *on* A*, with* $p_{a}>0$ *for all* a*. Suppose a coalition consisting of* c *manipulating agents among the* n *respondents of the current period—that is, of relative size* ρ=c/n∈(0,1]*—draws its answers from a manipulation distribution* $P_{man}\neq P_{ref}$*, while the remaining respondents draw from* $P_{ref}$*, so that the observed period distribution is the mixture* $P_{t}=\left(1-\rho \right)\;P_{ref}+\rho \;P_{man}$*. Then* $D_{KL}\left(P_{ref}∥P_{t}\right)>0$ *for every* ρ∈(0,1]*, and in a neighborhood of* ρ=0 *the divergence is strictly increasing and convex in* ρ*, with leading term *

$$D_{KL}\left(P_{ref}∥P_{t}\right)=\frac{\rho^{2}}{2ln2}\;\chi^{2}\left(P_{man},P_{ref}\right)+o\left(\rho^{2}\right),\;\;\chi^{2}\left(P_{man},P_{ref}\right)=\sum_{a\in A}\frac{{\left(m_{a}-p_{a}\right)}^{2}}{p_{a}},$$

*where* $m_{a}=P_{man}\left(a\right)$ *and* $\chi^{2}$ *denotes the Pearson chi-square divergence. The proof is presented in Appendix A.2*.

**Proposition** **3** (Reference entropy band)**.** *Under honest and interannually stationary responding, the plug-in entropy* ${\hat{H}}_{t}$ *of item* q*, computed from* $N_{t}$ *independent observations drawn from* $P_{ref}$*, is asymptotically Gaussian around* $H\left(P_{ref}\right)$ *and lies within the band* $\left[{LCL}_{q},\;{UCL}_{q}\right]=H_{ref}\pm \kappa \;\sigma_{H}$ *with probability approximately* $2\Phi \left(\kappa \right)-1$*—about* 0.954 *for* κ=2*—where* Φ *denotes the cumulative distribution function of the standard normal distribution* $N\left(0,1\right)$*. Conversely, under a manipulated mixture* $P_{t}=\left(1-\rho \right)\;P_{ref}+\rho \;P_{man}$ *with* ρ>0*, the expected entropy shifts to* $H\left(P_{t}\right)\neq H\left(P_{ref}\right)$*, and* ${\hat{H}}_{t}$ *exits the band, with probability tending to one once the shift exceeds* $\kappa \;\sigma_{H}$*. A departure from the band therefore indicates that the natural information structure of the distribution has been disrupted. The proof is presented in Appendix A.3*.

KL divergence functions in this study as a metric for the information distance between the expected and the observed. Let $P_{ref}$ be the reference distribution estimated from historical survey data. The value $D_{KL}(P_{ref}∥P_{t})$ calculated at time t is updated with each new response block. The exceedance of statistical control limits by this value indicates that the current distribution has meaningfully diverged from the reference structure, and points to potential manipulation.

For a symmetric comparison between distributions, the Jensen–Shannon divergence is preferred. Let $P_{ref}$ and $P_{t}$ be probability mass functions on A and define the mixture distribution $M=1/2\left(P_{ref}+P_{t}\right)$, which is itself a probability mass function on A. Since $M\left(x\right)>0$ whenever $P_{ref}\left(x\right)>0$ or $P_{t}\left(x\right)>0$, both $P_{ref}\ll M$ and $P_{t}\ll M$ hold by construction, so the two Kullback–Leibler terms in Equation (3) are always finite. The Jensen–Shannon divergence is then given by Equation (3):

(3) $$JSD\left(P_{ref}∥P_{t}\right)=1/2\;D_{KL}\left(P_{ref}∥M\right)+1/2\;D_{KL}\left(P_{t}∥M\right)$$

This metric evaluates two-way deviations with equal weight.

The literature fields examined in the previous subsections form the theoretical foundation of this study. While each field offers unique contributions, they possess structural limitations. Table 1 outlines these contributions and limitations.

This study fills the gap in question with an integrated approach. It inherits the data reliability problem from the survey quality literature, adopts the strategic actor model from game theory, adapts the manipulation characterization from the data integrity literature, and takes the formal measurement framework from the entropy-based decision-making literature. It then transforms all these elements into a dynamic detection and decision-support system.

## 3. Dynamic Entropy Monitoring and Multi-Layered Decision-Support Framework

**Figure 1:**
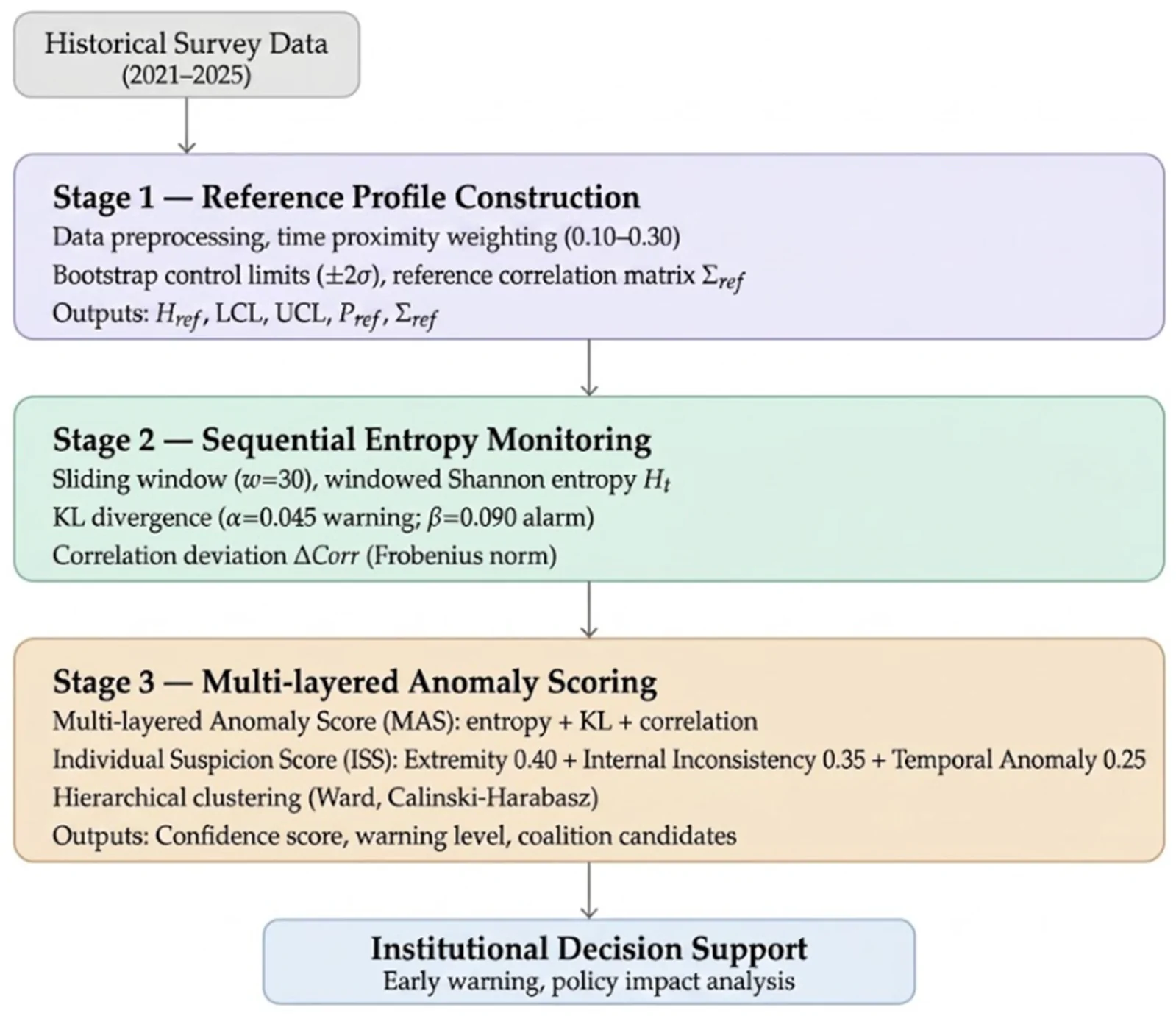
General flow diagram of the three-stage framework.

This section explains the operation of the three-stage dynamic entropy-monitoring framework in detail. In the first stage, the reference distribution and the baseline entropy profile are constructed. In the second stage, new survey responses are processed sequentially, and deviation signals are generated. In the third stage, a multi-layered anomaly scoring system is activated. Figure 1 visually summarizes this workflow. All parameters characterizing the framework, such as window size, weights, and thresholds, are determined by the calibration procedure explained in Section 3.4. The values adopted in the application of this study are stated next to the relevant equations.

### 3.1. Construction of the Reference Distribution and Baseline Entropy Profile

The objective of the first stage is to statistically define the expected response structure under conditions where no manipulation takes place. This structure is built over the reference distribution estimated from historical survey data.

Historical data consists of survey observations belonging to the years from $t_{-n}$ to $t_{-1}$. These data are passed through a three-step preprocessing workflow.

In the first step of data pre-processing, a data quality audit is performed. Observations with response times below the threshold are removed. Straight-lining profiles where a participant provides identical responses to all questions are detected. Participants whose missing data rate exceeds 20 percent are excluded from the analysis. The remaining missing values are completed using the multiple imputation method under the Missing Completely at Random assumption, which is denoted as MCAR.

In the second step, each observation is labelled according to the participant segment. Students, faculty members, and administrative staff are assigned to separate groups, allowing the analysis to be conducted at both the integrated and segment levels.

In the third step, each question is assigned to its respective dimension. This mapping ensures that entropy calculations can be performed at both the item and dimension levels.

Following the data pre-processing workflow, the reference distribution $P_{ref}\left(q,\;a\right)$ for each item q is calculated from the response frequencies across all reference years (Equation (4)):

(4) $$\;P_{ref}\left(q,\;a\right)=\;\frac{Number\;of\;participants\;responding\;\left\{from\;t_{-n}\;to\;t_{-1}\right\}}{Total\;number\;of\;participants\;\left\{from\;t_{-n}\;to\;t_{-1}\right\}}$$

In Equation (4), the numerator is the number of participants who selected response category a for item q, pooled across the reference years $t_{-n},\ldots ,t_{-1}$, and the denominator is the total number of participants who answered item q over the same reference years. $P_{ref}\left(q,a\right)$ is therefore the pooled relative frequency of category a for item q in the reference period. Since recent data provides a more reliable baseline, a time-weighted estimation is applied. The most recent year, $t_{\left\{-1\right\}}$, is assigned the highest weight. In the five-year application of this study, the weights are determined in an equally spaced manner, from the oldest year to the most recent year, as 0.10, 0.15, 0.20, 0.25, and 0.30. Dimension- and global-level distributions are calculated as the weighted average of the item distributions.

Shannon entropy is calculated for each reference year and each item q:

(5) $$H_{ref}\left(q,\;t\right)=-\sum_{a\in A}P\left(q,\;a,\;t\right)\cdot log_{2}\;P\left(q,\;a,\;t\right)$$

In Equation (5), q∈Q indexes the survey item and t indexes the reference year; A={1,…,5} is the Likert response space; $P\left(q,a,t\right)$ denotes the empirical probability (relative frequency) with which response category a was selected for item q by the participants of year t; and $H_{ref}\left(q,t\right)$ is the resulting Shannon entropy of item q in year t, expressed in bits. This calculation is repeated for n reference years to obtain the mean $\mu_{H,q}$ and the standard deviation $\sigma_{H,q}$ for each question. The same process is applied at the dimension and global levels.

Statistical control limits are derived from these values. In the general formulation, control limits are defined as a confidence band around the mean, as shown in Equations (6) and (7):

(6) $$UCL_{q}=\mu_{H,q}+\kappa \;\sigma_{H,q}$$

(7) $$LCL_{q}=\mu_{H,q}-\kappa \;\sigma_{H,q}$$

In classical statistical process control, κ is set to 3. However, in institutional survey environments where the number of reference years is limited, the estimation of $\sigma_{H,q}$ obtained from interannual variance can be noisy. Therefore, in this study, control limits are calculated via bootstrap resampling instead of the direct interannual standard deviation. This part of the methodology is detailed further in Section 3.4.

In this approach, a bootstrap distribution with 1000 iterations is generated for each dimension, and control limits are determined as plus or minus 2 times the standard deviation of this distribution, which corresponds to κ=2 and a confidence level of approximately 95.4 percent. These limits define the statistical boundaries of the natural entropy band and constitute the empirical equivalent of Proposition 3, stated in Section 2.3.

The natural correlation structure among the items in the survey is defined through the reference correlation matrix $\sum_{ref}$. This matrix is utilized in coordinated coalition detection. Coordinated coalition behavior tends to disrupt the natural correlation structure, and this disruption is measured via the Frobenius norm in subsequent stages.

### 3.2. Sequential Entropy Monitoring and Divergence-Based Anomaly Detection

In the second stage, the new survey period begins. Entropy and KL divergence are updated as responses arrive. This approach carries the capacity to generate anomaly signals before data collection is fully completed. In treating each incoming block as a potential distributional change relative to a historically estimated reference, the procedure connects to the broader information-criteria literature on change-point detection [42], which it adapts to the institutional survey setting.

Incoming responses are sorted according to their initial timestamps and then processed in sliding windows of size w. The window advances by one step with each new block of responses. The window size w is a critical parameter balancing statistical power and response speed. Small values for w provide an early warning but increase the risk of false positives. Conversely, large values for w produce more stable estimations but increase the warning latency. In the application of this study, w=30 participants were selected, in accordance with the calibration procedure described in Section 3.4.

Cumulative entropy and windowed entropy are calculated in parallel. While the cumulative calculation monitors the general trend, the windowed calculation captures instantaneous fluctuations. Entropy is calculated from the response distribution within the w-window at time t using Equation (8):

(8) $$H_{t\left(q\right)}=-\sum_{a\in A}P_{t\left(a\;|\;q\right)}\cdot log_{2}\;P_{t\left(a\;|\;q\right)}$$

In Equation (8), $P_{t}\left(a|q\right)$ denotes the empirical conditional distribution of responses to item q within the sliding window at step t; that is, $P_{t}\left(a|q\right)$ is the relative frequency of category a∈A among the responses to item q given by the w participants contained in the current window. $H_{t}\left(q\right)$ is the corresponding windowed Shannon entropy of item q at step t, expressed in bits. This value is calculated at each window step and the resulting time series $H_{t\left(q\right)}$ is compared with the reference control limits. Three states are defined. In the case where $H_{t\left(q\right)}$ is less than $LCL_{q}$, an artificial consensus alarm is generated, pointing to Type I or Type II manipulation. In the case where $H_{t\left(q\right)}$ is greater than $UCL_{q}$, an alarm indicating that the natural structure of the distribution is disrupted is generated. This second case can be a sign of both random responses, Type III, and coalition behavior, Type IV, where coordinated manipulation inflates the tails of the reference distribution. The system operates normally when $H_{t\left(q\right)}$ remains between $LCL_{q}$ and $UCL_{q}$. Additionally, entropy momentum is calculated as $\Delta \;H_{t}=H_{t}\;-\;H_{t-1}$ to capture sudden shifts. Large drops or surges occurring within a single window generate an additional warning signal.

The information distance between the current distribution and the reference distribution is calculated at each window step, using Equation (9):

(9) $$\;D_{KL,t}\left(q\right)=\sum_{a\in A}P_{ref}\left(a|q\right)\;{log}_{2}\;\left[\frac{P_{ref}\left(a|q\right)}{P_{t}\left(a|q\right)+\varepsilon }\right]$$

In Equation (9), $P_{ref}\left(a|q\right)$ is the reference probability of response a to item q obtained in Stage 1 (Equation (4)); $P_{t}\left(a|q\right)$ is the windowed empirical distribution defined for Equation (8); and ε is a Laplace correction that prevents the denominator from becoming zero, set to ε=1/N, where N represents the total number of reference observations. The resulting value $D_{KL,t}\left(q\right)$ is compared against two thresholds, where exceeding the α threshold produces a yellow warning and exceeding the β threshold generates a red alarm. The condition α < β is always valid. In the application of this study, α=0.045 and β=0.090 were determined according to the calibration procedure. The dimension-level KL divergence is calculated as the weighted average of the question-level values, and the global KL divergence is computed using the same method across all dimensions. These empirical quantities are the sample counterparts of the population objects analyzed in Propositions 1–3. Under honest responding, the responses inside the window are independent draws from the reference distribution $P_{ref}\left(\cdot |q\right)$, so $H_{t}\left(q\right)$ in Equation (8) is the plug-in estimator of $H\left(P_{ref}\right)$ whose concentration within the band $\left[{LCL}_{q},{UCL}_{q}\right]$ is established by Proposition 3. Under coordinated manipulation of relative intensity ρ, the window distribution converges to the mixture $P_{t}=\left(1-\rho \right)\;P_{ref}+\rho \;P_{man}$ analyzed in Proposition 2, and $D_{KL,t}\left(q\right)$ in Equation (9) is the empirical estimate of the strictly positive divergence $D_{KL}\left(P_{ref}∥P_{t}\right)$, whose growth in ρ Proposition 2 quantifies; the Laplace correction ε plays the role of the absolute-continuity safeguard discussed below Equation (2). Finally, the detection probability $\pi \left(\delta \right)$ induced by these monitoring statistics is the leader-side instrument of the Stackelberg deterrence result in Proposition 1.

The current correlation matrix $\Sigma_{t}$ is computed at each window step and the deviation from the reference matrix is measured using the Frobenius norm, as shown in Equation (10):

(10) $$\Delta {Corr}_{t}={∥\Sigma_{t}-\Sigma_{ref}∥}_{F}$$

In Equation (10), $\Sigma_{t}$ denotes the inter-dimensional correlation matrix estimated from the responses in the current window; $\Sigma_{ref}$ the reference correlation matrix constructed in Stage 1; and ${∥\cdot ∥}_{F}$ the Frobenius norm, defined for a real matrix $M=\left(m_{ij}\right)$ as

$$\;{∥M∥}_{F}=(\sum_{i}\sum_{j}m_{ij}^{2}{)}^{1/2},$$

that is, the square root of the sum of the squared entries. $\Delta {Corr}_{t}$ therefore aggregates the elementwise deviation between the current and reference correlation structures into a single non-negative scalar. This metric is associated with the detection of Type IV coordinated coalition behavior. While individual manipulation disrupts the correlation structure in a limited manner, a coordinated coalition systematically alters correlations across specific dimensions. A high value for $\Delta Corr_{t}$ points to this second situation.

### 3.3. Multi-Layered Anomaly Scoring System

In the third stage, the three outputs of Stage 2, which are entropy deviation, KL divergence, and correlation disruption, are integrated into a single composite score. This process is executed at both the population and individual levels.

The Multi-Layered Anomaly Score for dimension q at time t is calculated through Equation (11):

(11) $$\;MAS_{t}(q)=w_{1}\cdot \;norm\left[z_{\Delta \;H(t,q)}\;\right]+\;w_{2}\cdot \;norm\left[D_{KL,t}(q)\right]+\;w_{3}\cdot norm\left[\Delta Corr_{t}\right]$$

In this formulation, $z_{\Delta \;H(t,q)}$ represents the standard score of the entropy momentum computed from the reference distribution. The term $norm[D_{KL,t}(q)]$ signifies the KL divergence value normalized to a range between 0 and 1, and $norm[\Delta \;Corr_{t}]$ denotes the normalized value of the correlation disruption. To keep the three components on a common scale, each term in Equation (11) is min–max normalized to the [0, 1] interval prior to aggregation; norm[·] denotes this normalization, which is applied also to the absolute entropy-momentum score $\left|z_{\Delta \;H(t,q)}\right|$. The calibration procedure of Section 3.4 yielded $w_{1}=0.35$ for the entropy-momentum term, $w_{2}=0.45$ for the KL-divergence term, and $w_{3}=0.20$ for the correlation-disruption term, subject to $w_{1}+w_{2}+w_{3}=1$. The KL term receives the largest weight, because it is the earliest and strongest indicator of coordinated coalition entry; the entropy term is second, as it localizes dimension-selective distortion; and the correlation term is assigned the lowest weight, owing to its higher estimation variance in short windows. The global anomaly score is computed as the geometric mean of the dimension-level MAS values, which prevents an extreme deviation in a single dimension from dominating the global indicator. A global score exceeding $\tau_{1}=0.50$ produces a yellow warning, while a value surpassing $\tau_{2}=0.70$ generates a red alarm, with $\tau_{2}\;>\;\tau_{1}$ always maintained.

Individual analysis is activated when a population-level anomaly is detected. For each participant i, the Individual Suspicion Score is calculated via Equation (12):

(12) $$\;ISS_{i}=w_{a}\cdot \;{ExtScore}_{i}+\;w_{b}\cdot \;{Intconsistency}_{i}+\;w_{c}\cdot \;{TempAnomaly}_{i}$$

The specific metrics constituting this composite score are defined below:

- Extremity Score (ExtScore): This metric measures the position of the response given by the participant to each question within the population distribution. Responses falling into the bottom or top five-percent slice of the reference distribution are classified as outliers, and the proportion of such responses determines the overall extremity score for the individual.
- Internal Inconsistency (Intconsistency): This component evaluates the response variance across questions belonging to the exact same dimension. Selecting mutually exclusive extreme values within a single dimension is recorded as an inconsistency. A high inconsistency score indicates that the responses lack informational value or are generated via a random mechanism.
- Temporal Anomaly (TempAnomaly): This value is computed when precise timestamp records are accessible. If the total survey completion time of a participant deviates substantially from the median duration established by other respondents, the agent is flagged. The system evaluates both exceptionally rapid and unusually prolonged completion patterns.

The component weights, where $w_{a}=0.40$, $w_{b}=0.35$, and $w_{c}=0.25$, are established based on a rationale rooted in theoretical priority ranking. The extremity score and internal inconsistency components directly evaluate the response content, thereby possessing the most direct causal connection to manipulation behavior. Consequently, these content-based metrics receive 75 percent of the total weight allocation. The temporal anomaly component is assigned a lower weight due to its dependency on timestamp data quality.

This initial configuration provides a stable baseline for future optimization routines utilizing a validation dataset with ground-truth labels. The Monte Carlo experiments presented in Section 5.5 confirm that this weight configuration yields a robust and consistent detection performance across various manipulation intensities. In scenarios where timestamp data is unavailable, the score is computed using the remaining two components, and the weights are re-normalized accordingly.

The determination of these weights and the extent to which their choice affects the results warrant explicit discussion. The weights enter the Individual Suspicion Score linearly; consequently, small perturbations of the weight vector translate into proportionally small changes in the composite score and leave the ranking of clearly flagged participants unchanged. The extent to which the weight choice can affect the results admits a direct analytical bound. Since each component score lies in [0, 1] and the ISS is linear in the weights, replacing the baseline vector (0.40, 0.35, 0.25) by any perturbed vector within ±0.10 of it changes no participant’s composite score by more than 0.10; a perturbation of this magnitude cannot reorder participants whose baseline scores differ by more than this bound, so the flagging decisions for clearly separated participants are invariant over the entire perturbation range. Detection of Type I and Type II manipulation is entirely insensitive to the weight choice, because these types are captured by the deterministic straight-lining filter that operates upstream of the ISS. For Type III and Type IV, Section 5.5 shows that the weak individual-level separability arises from structural properties of the manipulation types themselves—the lack of directionality in noise injection and the dimension-selective mimicry of coalition members—which no reweighting of the same three components can overcome; the qualitative conclusions are therefore not artifacts of the particular weight vector. An analogous robustness assessment applies to the remaining parameters of the framework. The window size w, the KL thresholds α and β, the control-limit coefficient κ, and the population-level weights $w_{1}$, $w_{2}$, $w_{3}$ are selected jointly by the cross-validated calibration of Section 3.4 under the institutional false-positive constraint (FPR≤5%), and the parameter sensitivity analysis embedded in that procedure confirms that the selected combination lies on a performance plateau rather than a knife-edge. Among all parameters, detection behavior is most sensitive to the window size w, which directly governs the latency–variance trade-off discussed in Section 3.2, whereas the thresholds α, β, $\tau_{1}$, and $\tau_{2}$ shift the precision–recall balance monotonically and can be tightened during high-stakes decision periods, as noted in Section 3.4.

Detection at the individual level directly complements the coordinated coalition analysis. Participants exhibiting a high score are subjected to a secondary evaluation via clustering algorithms. Hierarchical clustering is applied utilizing the Ward linkage method, focusing on response pattern similarities. The optimal number of clusters is determined dynamically via the Calinski–Harabasz index, which monitors the ratio of intra-cluster to inter-cluster variance.

Each detected cluster is evaluated against two primary operational criteria:

- Cluster Homogeneity: This metric quantifies the similarity of response profiles among individuals within the same cluster, where high uniformity serves as a strong signal of coordinated action.
- Temporal Concentration: This parameter measures the proximity of survey entry timestamps for individuals grouped in the same cluster. Members of a coordinated coalition typically exhibit a tendency to access the system within narrow time windows.

Clusters that simultaneously exceed the established thresholds for both criteria are flagged as definitive coalition candidates, and these groups are subjected to further specialized interpretation within the institutional framework.

### 3.4. Threshold Calibration and Statistical Control Limits

Five primary parameter groups of the framework require calibration. These groups consist of the window size denoted as w, the population-level score weights represented by $w_{1},\;w_{2},\;w_{3}$, the individual score weights given by $w_{a},\;w_{b},\;w_{c}$, the warning thresholds written as α and $\tau_{1}$, and the alarm thresholds specified as β and $\tau_{2}$.

Calibration is performed via cross-validation over the reference data. The reference years are divided into k folds. Each time, one fold is reserved as the validation set while the remaining folds serve as training data. Controlled synthetic manipulation is injected into this training data and the false positive rate, along with the false negative rate, are calculated for every parameter combination.

The institutional false positive tolerance is set to a false positive rate less than or equal to 5 percent. The parameter combination that maximizes the F1 score under this constraint is selected. Parameter sensitivity analysis verifies the robustness of the selected combination. As a result of this procedure, the values adopted in the application of this study include a window size w=30, KL thresholds α=0.045 and β=0.090, a control limit coefficient κ=2 derived via bootstrap, and individual score weights $w_{a}=0.40,\;w_{b}=0.35,\;w_{c}=0.25$.

The institutional context is also considered when determining thresholds. During high-risk decision periods such as strategic plan revisions or budget allocations, thresholds can be lowered to make the system more sensitive. This flexibility ensures the integration of the framework into the institutional decision calendar.

### 3.5. Computational Complexity and Applicability of the Framework

The computational complexity of the framework is efficient. The reference profile calculation is performed only once. Sequential monitoring possesses a complexity of O(m.k), where m denotes the number of items and k represents the number of Likert categories. The individual score calculation has a complexity of O(n.m), where n is the number of participants. Hierarchical clustering exhibits a complexity of $O(n^{2})$ and executes only when a population-level alarm is generated.

On a standard university survey scale, where n is approximately 1000, m=16, and k=5, all calculations are completed within seconds. This situation supports the applicability of the framework as a real-time early warning system. The framework does not require labelled training data. The system can be deployed directly if historical survey records are available and participant segmentation is known. This feature offers a critical practical advantage for institutional adoption. Table 2 summarizes the inputs, core calculations, and outputs of the three stages.

## 4. Empirical Application

This section applies the first stage of the dynamic entropy-monitoring framework developed in Section 3 to actual corporate data. Five years of stakeholder survey data are subjected to a systematic pre-processing procedure to establish a robust reference entropy profile and lay the groundwork for monitoring subsequent survey periods based on this profile. The outputs of this stage form the reference framework for the validation conducted on a test set involving synthetic manipulation in Section 5.

### 4.1. Dataset and Institutional Context

The application was conducted on academic staff satisfaction survey data from a public university covering the years 2021 to 2025. These surveys are regularly administered within the scope of the annual evaluation cycle of the institution, and participant responses are automatically recorded by the system. In compliance with the principle of institutional anonymity, participant-identification details were anonymized before the data were made available for research.

The nature of the dataset must be explicitly stated for methodological transparency. The entire five-year observation series for the years 2021 to 2025 consists of actual corporate survey records maintained in the digital survey archive of the respective public university and anonymized in accordance with international research ethics standards. These five-year data are utilized to establish the reference pool of the framework. To validate the detection capacity of the framework under controlled conditions a test set for the 2026 period was also generated. This test set was constructed, via Monte Carlo simulation, based on the distribution parameters of the five-year reference profile, and was designed to include four distinct types of manipulation at known intensities. The design and validation findings of the test set are presented in detail in Section 5. The distribution parameters underlying the reference profile are reported in Section 4, and the response models and intensity settings used to generate the synthetic test data are specified in Section 5.1.

The survey consists of five dimensions, comprising Management Leadership and Belonging, Education and Training, Research and Development, Social Contribution, and Working Environment, and includes 16 items. The distribution of items per dimension is as follows: four items for Management Leadership and Belonging, four items for Education and Training, three items for Research and Development, three items for Working Environment, and two items for Social Contribution. Responses were collected using a 1 to 5 Likert scale. The start and end dates of the survey are recorded for each participant. These timestamps are utilized for temporal anomaly detection in subsequent analyses.

The dataset is structured in the long format, where each observation represents the response of a participant to a specific question. Table 3 summarizes the overall structure of the dataset on an annual basis in its raw state prior to pre-processing.

Regarding the raw data, the number of participants demonstrates a continuous increase over the years. The average annual growth rate in the number of participants between 2021 and 2024 is 5.9%. A distinct surge of 9.7% is prominent in 2025. A relatively sharp rise in the raw mean compared to other years is also observed in the same period, corresponding to an increase of 0.197 points relative to 2024. These two patterns become more meaningful in light of the data quality assessment findings elaborated below.

### 4.2. Data Pre-Processing and the Empirical Motivation of the Study

As emphasized in Section 3.1, the reliability of the reference data itself directly determines the validity of all subsequent analyses. Therefore, the raw data are subjected to a robust pre-screening process. The most decisive component of this process is the detection of straight-lining. A participant providing identical responses to all questions creates a straight-lining profile. In a survey comprising five distinct dimensions and 16 items, the probability of this pattern reflecting a genuine and differentiated preference structure is statistically extremely low. This profile is the most explicit and easily detectable individual-level indicator of the strategic manipulation behavior defined in Section 2.2.

The straight-lining screening generated a critical finding that also reveals the empirical motivation of this study. As seen in Table 4, no participant exhibited a straight-lining profile during the four-year period covering 2021 to 2024. In contrast, six participants, corresponding to 2.1 percent, demonstrated a straight-lining profile for the first time in 2025. Five of these participants reported the highest value, 5, for all questions, while one participant reported the lowest value, 1, for all questions.

The nature and timing of this finding rather than its magnitude are methodologically decisive. The direct impact of the six straight-lining participants on the 2025 raw mean is limited. When straight-lining participants are excluded the 2025 mean decreases by only 0.015 points. Therefore, this pattern alone does not severely distort the reference pool. However, the primary significance of the finding is that it represents a signal of manipulation behavior that emerged from a stable baseline in the fifth year, after being completely absent for four years. As noted in the Introduction, this visible pattern represents only the most overt end of the strategic-response spectrum. Whether more subtle and coordinated forms of strategic behavior, defined as Type III and Type IV in Section 2.2, exist simultaneously in a manner undetectable by a simple screening cannot be directly observed. In an institutional context where survey results directly feed into resource allocation program evaluation and strategic planning decisions, this visible signal concretely demonstrates the need for a systematic monitoring capacity. The dynamic entropy-monitoring framework developed in this study responds precisely to this need.

Following the pre-processing stage, the five-year data were transformed into a clean reference pool by removing straight-lining profiles. The clean consolidated reference pool consists of 19,728 observations belonging to 1233 participants. No observations were removed during the 2021 to 2024 period, while six straight-lining participants comprising 96 observations were separated from the pool in 2025. All subsequent analyses are conducted on these clean data.

### 4.3. Annual Entropy Trend

The Shannon entropy, defined in Section 3.1, was calculated for each year and each dimension, using the clean data. Table 5 presents the entropy trend at both the dimensional and global levels.

**Table 5:** Year × dimension entropy matrix for clean data, base log_2._

Dimension	2021	2022	2023	2024	2025
Management Leadership and Belonging	2.2724	2.2417	2.2372	2.2327	2.1717
Education and Training	2.2373	2.1623	2.1733	2.1651	2.0953
Research and Development	2.2166	2.2363	2.2062	2.1710	2.1227
Social Contribution	2.2205	2.2045	2.1632	2.1248	2.0364
Working Environment	2.2720	2.2569	2.2459	2.2504	2.2270
Global	2.2647	2.2388	2.2291	2.2199	2.1545

Note. The entries in Table 5 are the annual Shannon entropies $H\left(d,t\right)$ of dimension d in year t, computed from the clean data of that single year; they are not yet the combined reference values $H_{ref}$ reported in Table 6.

**Table 6:** Dimension-level combined reference profile.

Dimension	$H_{ref}$	LCL	UCL	$P_{ref}$ Mean
Management Leadership and Belonging	2.2206	2.2049	2.2364	3.405
Education and Training	2.1526	2.1334	2.1718	3.577
Research and Development	2.1779	2.1572	2.1986	3.497
Social Contribution	2.1275	2.0948	2.1603	3.670
Working Environment	2.2456	2.2300	2.2612	3.207

Note. Table 6 is derived from the annual quantities underlying Table 5: $H_{ref}$ is obtained by combining the annual response distributions with the time-proximity weights 0.10, 0.15, 0.20, 0.25, and 0.30 for 2021–2025, and the control limits LCL and UCL are computed from 1000 bootstrap resamples of this weighted combination (±2 standard deviations of the bootstrap distribution). The annual values of Table 5 are thus the inputs from which the combined reference profile of Table 6 is constructed.

Two main findings stand out. First, entropy exhibits a downward trend, across all dimensions, from 2021 to 2025. The entropy decrease between 2021 and 2025 at the global level is 0.1102 units. A significant portion of this decrease is concentrated in the 2024 to 2025 transition, corresponding to 0.0654 units, which coincides with the raw mean surge and the initial appearance of straight-lining behavior in 2025. Nevertheless, all values remain in the high-diversity band close to the theoretical upper limit of the scale, which is $H_{max}=log_{2}\left(5\right)=2.3219$. Second, the Working Environment dimension remains relatively isolated from this downward trend. This dimension maintains the highest and most stable entropy level over the five years, within the range of 2.2720 to 2.2270. This situation indicates that participants’ evaluations of working conditions exhibit a more heterogeneous and stable structure compared to other dimensions.

### 4.4. Combined Reference Entropy Profile

Directly combining five years of data introduces the risk of the early years excessively influencing the current structure. Time proximity weighting is applied to mitigate this risk. The weights are determined as 0.10 for 2021, 0.15 for 2022, 0.20 for 2023, 0.25 for 2024, and 0.30 for 2025. This weighting prioritizes recent experience, ensuring that the reference profile reflects current institutional conditions. A bootstrap sampling with 1000 iterations is applied across each dimension to quantify the uncertainty of $H_{ref}$ values. During each bootstrap iteration, responses are resampled with replacement on an annual basis and the annual entropies are combined using the same time proximity weights. Control limits are calculated as plus or minus two standard deviations of the bootstrap distribution. This selection corresponds to a 95.4 percent confidence level. Table 6 presents the dimension-level combined reference profile.

The five-year combined structure significantly narrows the control bands compared to a single-year reference. For instance, when bootstrapping is performed exclusively with the 2025 data in the Management Leadership and Belonging dimension, the width between LCL and UCL is 0.0755 units, whereas this width decreases to 0.0315 units in the five-year combined reference. The narrower band increases the statistical power in distinguishing genuine deviations from noise.

The $H_{ref}$ values of all dimensions are concentrated between 2.13 and 2.25. This outcome reveals that institutional survey response distributions naturally form a stable region that is close to $H_{max}=2.3219$ but remains distinctly below it. Proposition 3, stated in Section 2.3, is supported by this empirical band.

### 4.5. Global Reference Profile

The global response distribution from 1 to 5, calculated over the combined clean reference data, using time proximity weights, was obtained respectively as follows: 0.1300, 0.1016, 0.2090, 0.2962, and 0.2633. The global $H_{ref}$ was calculated as 2.2166 from this distribution. The normalized $H_{ref}$ value, calculated by dividing $H_{ref}$ by $H_{max}$, is 0.9546. The global reference mean is 3.461.

This distribution reflects a structure where responses 4 and 5 are predominant, with a combined share of 55.9%, yet a distinct diversity of opinion is preserved. The global mean of the raw data, being 3.434, remains close to the clean reference mean of 3.461. The minor difference between them originates from the weighting method and the straight-lining clean-up. Here the global $H_{ref}$ is computed as the entropy of the pooled time-weighted global distribution; by the concavity of entropy, this slightly exceeds the time-weighted average of the annual global entropies (≈2.209).

### 4.6. Reference Correlation Structure

The interdimensional correlation matrix calculated over the five-year clean data is presented in Table 7. This matrix constitutes the reference point for the calculation of $\Delta {Corr}_{t}={∥\Sigma_{t}-\Sigma_{ref}∥}_{F}$ (Equation (10)), which will be utilized for coordinated coalition detection in subsequent survey periods. The correlations were computed based on the dimension-level mean scores of each participant.

Interdimensional correlations range between 0.259 and 0.362. The correlation between the Working Environment and the Management Leadership and Belonging dimensions attains the highest value, with an r of 0.362. Conversely the correlation between Social Contribution and Working Environment remains at the lowest level, at r equal to 0.259. This moderate positive correlation structure indicates that, although the dimensions share a common institutional satisfaction trend, they function as diverging independent evaluation axes.

When compared to the single-year reference, where the r range for 2025 is 0.644 to 0.805, the five-year combined correlations, with an r range of 0.259 to 0.362, are observed to be at distinctly more moderate levels. This difference stems from the integration of heterogeneous response structures spanning multiple years. Since the five-year $\Sigma_{ref}$ represents a broader institutional variation, it provides a more conservative and reliable baseline for correlation anomaly detection in future survey periods.

### 4.7. Summary of Stage 1 Reference Parameters

The five-year data processing procedure generated all reference parameters required by the monitoring system for Stage 2 and Stage 3. Table 8 summarizes these outputs.

The five-year combined reference structure provides three fundamental advantages compared to a single-year design. First the total of 19,728 clean observations significantly narrows the bootstrap confidence intervals and enhances the capacity of the control limits to reflect genuine variation. Second the five-year trend analysis reduces the risk of a single year being an anomalous period in determining the natural entropy band. Third, narrower control bands facilitate the differentiation of genuine anomalies from noise.

Regarding the sustainability of the framework, it is recommended to incorporate the post-pre-processing clean data into the reference pool and update the time proximity weights upon the completion of each new survey period. This dynamic update mechanism will prevent the reference profile from becoming obsolete over time by tracking institutional development.

Nevertheless, a limitation must be explicitly noted. It cannot be directly known whether more subtle forms of the straight-lining pattern observed for the first time in 2025, such as coordinated but non straight-lining strategic responses, existed in previous years. This limitation necessitates the consistent application of the systematic pre-processing procedure in each period and the continuous monitoring of the holistic entropy values of the reference years for interannual consistency. The detection of zero straight-lining in four out of five years is a positive indicator supporting the framework assumption that the reference pool is largely free of manipulation.

## 5. Decision-Support Architecture and Validation

This section fulfills two primary functions simultaneously. First the decision-support architecture explaining how the framework developed in Section 3 will be integrated into institutional decision processes is presented. Second the detection capacity of the framework is empirically validated by applying said architecture on a controlled test set containing four distinct types of manipulation at known intensities.

The test data utilized for validation is a controlled observation set that strictly aligns with the actual institutional survey structure, while the composition of the manipulation groups is known in advance. This ground truth information enables the evaluation of detection findings against a definitive external criterion.

### 5.1. Design of the Test Dataset

The test dataset consists of 4768 observations from 298 participants for the 2026 period. It was generated, via Monte Carlo simulation, based on the distribution parameters of the five-year combined reference profile established in Section 4. Honest participants were sampled from the reference question distributions while manipulation groups were designed in accordance with the strategic response typology defined in Section 2.2. Participants are divided into six groups. Table 9 summarizes the group structure and manipulation types.

The total number of suspicious participants is 78, corresponding to 26.2 percent. This ratio represents a high-intensity manipulation scenario that could be observed in actual institutional environments.

The manipulation groups are designed as carrying out two distinguishing features of coordinated behavior, namely, temporal clustering and dimension-selective targeting. The 22 members of the Coordinated Suppression group are clustered in a narrow window in their initial timestamps, and these participants entered the system in the last third of the data collection period. This group reports a response of 1 with approximately 65 percent probability and a response of 2 with 35 percent probability to the target dimension of Management Leadership and Belonging, while producing responses conforming to the reference distribution for other dimensions. The 10 members of the Coordinated Inflation group enter the system in the very last window of data collection within an even narrower interval and report a response of 5 with approximately 70 percent probability and a response of 4 with 30 percent probability to the Research and Development dimension. This selective targeting is the defining characteristic of Type IV manipulation.

### 5.2. Stage 1 Application: Straight-Lining Detection

During the data preprocessing stage, 32 participants were flagged as straight-lining profiles. This number corresponds to 10.7 percent of the total participants. Of the flagged profiles, 28 reported the highest value of 5 for all questions while four reported the lowest value of 1.

Straight-lining detection exhibited perfect performance. All 32 instances of the Type I and Type II manipulation groups were correctly identified without any observed false positives or false negatives. Precision Recall and the F1 score each attained a value of 1.000. This finding demonstrates that the standard deviation-based pre-screening procedure defined in Section 3.1 functions as a reliable filter for this type of manipulation.

The impact of straight-lining participants on the raw mean is distinct. The raw mean of the test set is 3.521, and when straight-lining participants are excluded, this value decreases to 3.403. The difference of 0.118 points corresponds to 2.4 percent of the 5-point Likert scale. This difference indicates that institutional evaluation decisions based on raw data can be systematically misled.

### 5.3. Stage 2 Application: Sequential Entropy Monitoring

After filtering out straight-lining profiles, Shannon entropy was calculated on a dimension basis for the remaining 266 participants and compared with the reference control limits obtained from Section 4. Table 10 presents this comparison.

Anomalies were detected in two dimensions, and these two dimensions are precisely the ones targeted by coordinated manipulation. The Management Leadership and Belonging dimension exceeded the UCL limit, with H=2.2562 > UCL=2.2364. The provision of only 1 and 2 responses by the Coordinated Suppression group inflates the tail regions of the distribution that was concentrated toward 4 and 5 during the reference period and thus artificially flattens the distribution, thereby increasing entropy. The Research and Development dimension also exceeded the UCL, with H=2.2106 > UCL=2.1986. The direction of this shift requires explanation, since concentrating responses on high categories does not always raise entropy. In the reference profile, the R&D modal category is 4, while category 5 carries a lower probability mass. The Coordinated Inflation group reports 5 with ~0.70 probability and 4 with ~0.30 probability, which lifts the under-represented category 5 toward the modal category 4 rather than sharpening an existing peak. As a worked illustration, with a reference R&D distribution of $P_{ref}=(0.09,\;0.12,\;0.23,\;0.34,\;0.22)$ over the 1–5 scale (mean 3.48, H=2.178, matching Table 6) and 10 inflation members among 266 respondents, the resulting mixture distribution flattens the upper tail by raising P(5) relative to P(4); given the narrow reference band ($UCL\;-\;H_{ref}\;\approx \;0.02$), this is sufficient to push the windowed entropy above the UCL. Thus, in contrast to the radical homogenization produced by Type I/II straight-lining (which lowers entropy), soft Type IV inflation directed at an under-weighted upper category increases entropy by partially equalizing the upper tail. The three dimensions not subjected to manipulation, namely, Education and Training, Social Contribution, and Working Environment, remained within the reference band. This result demonstrates that entropy-based monitoring can accurately localize dimension-selective coordinated manipulation.

**Figure 2:**
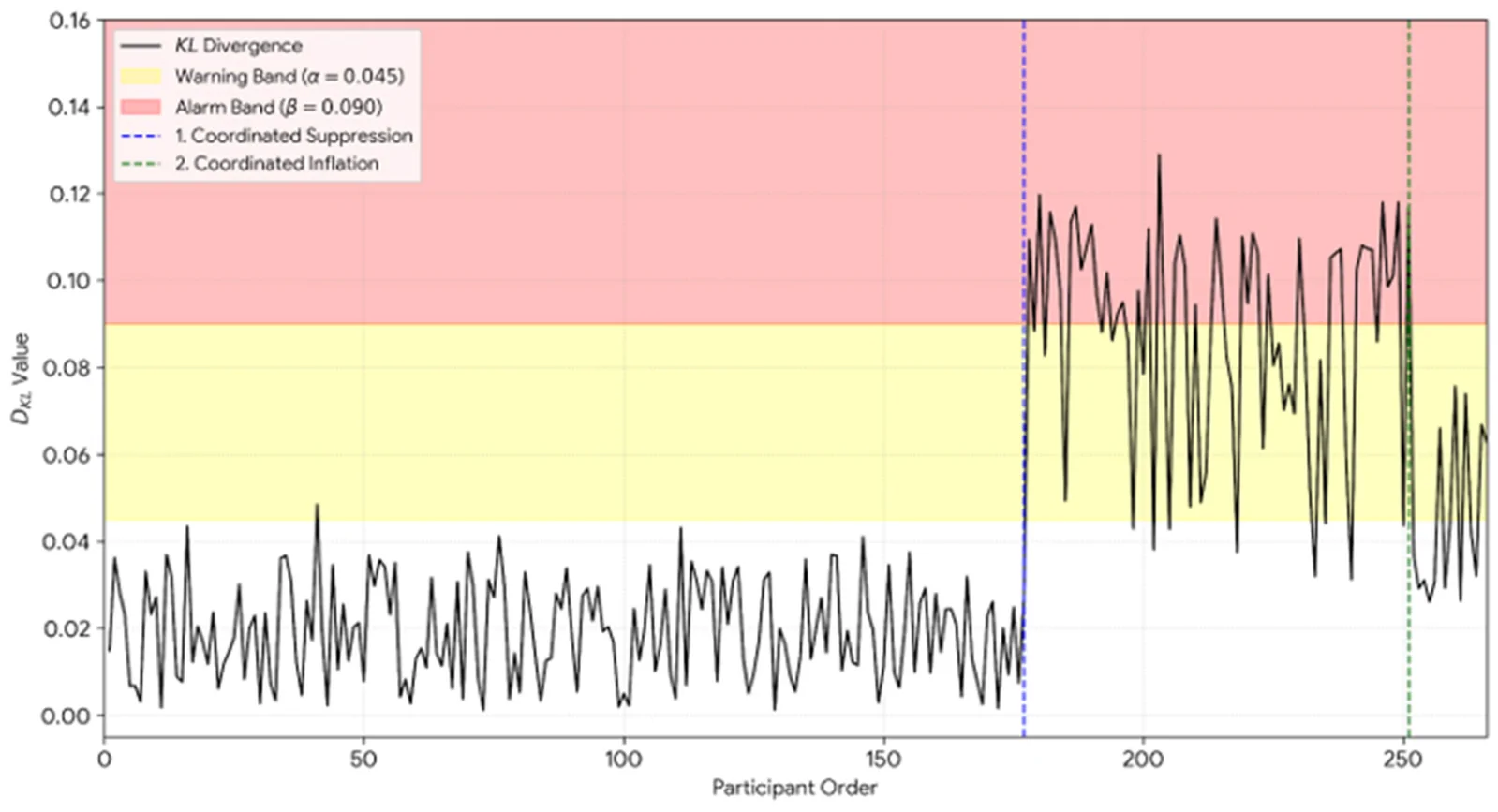
KL divergence time series.

After sorting the participants according to their initial timestamps, the global KL divergence was calculated using a rolling window of 30 participants. Figure 2 presents this time series. The thresholds were determined, as α=0.045 for the yellow warning and β=0.090 for the red alarm, in accordance with the calibration procedure.

Figure 2 illustrates the KL divergence time series, where the x axis represents the participant entry sequence and the y axis denotes the $D_{KL}$ value. The yellow band indicates the α=0.045 warning threshold while the red band represents the β=0.090 alarm threshold. Coalition entry windows are marked with vertical dashed lines.

Time-series analysis reveals distinctly diverging periods. In the first two-thirds of data collection, encompassing approximately the first 230 participants, KL values largely fluctuated within the 0.001 to 0.050 band, mostly remaining below the warning threshold. In the last third of the data collection period, accompanied by the intensive system entry of the Coordinated Suppression group, KL values rose sharply and reached the peak of the series at approximately 0.129. In this ascending region, KL values exceeded the red alarm threshold across a prolonged sequence of windows, and simultaneously, the intra-window Shannon entropy declined to the level of 2.01. At the very end of the series a secondary deviation region corresponding to the narrow entry window of the Coordinated Inflation group was observed. This pattern demonstrates that KL monitoring can capture coordinated coalition behavior simultaneously with the system entry windows of its members, which means before the completion of data collection.

### 5.4. Stage 3 Application: Multi-Layered Anomaly Scoring

The Individual Suspicion Score was calculated for the remaining 266 participants after the elimination of straight-lining profiles. The Individual Suspicion Score consists of the extremity score with a 40 percent weight, the internal inconsistency component with a 35 percent weight, and the temporal anomaly component with a 25 percent weight.

The distribution of the Individual Suspicion Score exhibited the following statistics, comprising a mean of 0.420, a median of 0.400, a standard deviation of 0.109, a minimum of 0.170 and a maximum of 0.772. The Individual Suspicion Score means on a group basis produced a methodologically critical result. While the mean Individual Suspicion Score of the Honest group is 0.417, the mean of the Coordinated Suppression group is 0.388, and the mean of the Coordinated Inflation group is 0.346. Thus, the individual mean scores of the coordinated coalition groups are not distinctively higher than those of the honest group and are in fact slightly lower. Only the Noise Injection group, with a score of 0.577, significantly diverges at the Individual Suspicion Score level, due to its high internal variance, but as will be demonstrated below, this divergence is insufficient for detection.

This finding is an expected and theoretically meaningful outcome. Members of the coordinated coalition exhibit deviation solely in a single target dimension and produce responses conforming to the reference distribution across all other dimensions. This selectivity renders the individual response profiles of the members holistically indistinguishable from honest participants. Therefore, the Individual Suspicion Score alone cannot differentiate coalition membership. This limitation directly demonstrates the necessity of the clustering analysis applied in the subsequent layer.

Hierarchical clustering was applied, using the Ward linkage method, over dimension-level response profiles. The optimal number of clusters was determined by evaluating the Calinski–Harabasz index and cluster interpretability together. Clustering powerfully revealed the coordinated pattern that the Individual Suspicion Score concealed on its own.

The cluster producing the strongest coalition signal is a 17-member cluster of which 13 belong to the actual Coordinated Suppression group, corresponding to a cluster purity of 76.5% (13/17) and a recall of 59.1% (13/22) for the suppression coalition. The cluster therefore isolates the majority of the coalition with low contamination, while the remaining nine members are recovered neither by individual scoring nor by clustering—a residual gap discussed in Section 6.3. The mean of the Management Leadership and Belonging dimension for the cluster is 1.46, which is approximately 1.95 points below the reference mean of 3.405 for the relevant dimension. The average survey completion time for the cluster members is 2.8 days, and their initial timestamps are concentrated in the final window of data collection. Both the high cluster purity and the distinct temporal clustering satisfy the two criteria necessary to flag this cluster as a coalition candidate.

In contrast the Coordinated Inflation group diverged only weakly in the clustering analysis. The application of a soft inflation predominantly using 4 and 5 on the Research and Development dimension targeted by this group causes the members to statistically overlap with honest participants who naturally assign high scores in Research and Development. This limitation demonstrates that the dimension-level entropy exceedance, namely the Research and Development UCL exceedance in Stage 2, provides a stronger detection signal for this group, relative to clustering. This situation highlights the importance of the layered design of the framework, once again demonstrating that distinct types of manipulation are captured at different layers.

### 5.5. Scenario Analysis

A controlled Monte Carlo experimental design was implemented to systematically determine the statistical operating characteristics of the framework. The experimental matrix consists of four manipulation types, five intensity levels, and 30 independent replications, resulting in a total of 600 scenarios. A dataset of 150 participants is generated in each scenario. Participants corresponding to the specified intensity ratio respond according to the relevant manipulation type, while the remainder produce honest responses from the reference distribution. Detection performance is measured via Precision Recall F1 score and AUC ROC metrics. The theoretical data-generating and decision model underlying Table 11 is as follows. In each scenario, honest participants draw their responses independently from the reference item distributions $P_{ref}\left(\cdot |q\right)$ estimated in Section 4 (multinomial sampling). Manipulating participants respond according to the type-specific response models of Section 2.2 and Section 5.1: Type I reports category 1 and Type II reports category 5 deterministically for all items; Type III draws uniformly at random from A for every item; Type IV draws from the coalition mixture concentrated on the low categories {1,2} with probabilities 0.65/0.35 (suppression) or on the high categories {5,4} with probabilities 0.70/0.30 (inflation) on the target dimension, and from $P_{ref}\left(\cdot |q\right)$ on all other dimensions. Participant-level detection combines the deterministic straight-lining filter of Stage 1 with the ISS-based flagging of Stage 3; Precision, Recall, F1, and AUC are then computed against the known ground-truth labels of each scenario and averaged over the 30 independent replications.

Table 11 presents the mean over the 30 replications for each metric; the dispersion across replications is characterised qualitatively below. For the Type I and Type II families the standard deviations are negligible (≤0.01), confirming that near-perfect detection is stable rather than driven by favorable replications; for the Type III and Type IV families the larger replication variability reflects the unreliability of individual-level detection for these types.

The findings reveal a distinct performance gradient across manipulation types. Type I and Type II manipulations yield a Recall close to 1.000 and an AUC value of 1.000 across all intensity levels due to the straight-lining detection mechanism. An almost perfect detection is achieved at all intensities for these types.

Type III Noise Injection hovers just around the random threshold, with AUC values remaining in the range of 0.490 to 0.526. This finding confirms that randomly responding participants statistically overlap with honest participants who naturally exhibit high variance on the Likert scale and that noise injection constitutes the structural limitation of the framework.

Type IV Coalition demonstrates only a limited improvement dependent on intensity at the individual level, exhibiting an AUC between 0.610 and 0.656. Individual scoring alone cannot reliably differentiate coalition membership. However as demonstrated in Section 5.4, the hierarchical clustering layer largely compensates for this limitation and captures the suppression coalition concealed by individual scoring with a cluster purity of 76.5 percent.

The detection traces left by the four manipulation types defined in Section 2.2 and their relative detection difficulty are summarized comparatively in Table 12.

Individual straight-lining behavior produced a 100 percent detection rate across all scenarios. It is statistically the most easily detectable manipulation type because it completely eliminates the anticipated response diversity. Noise injection is the most difficult type to distinguish from natural variation, in terms of both Individual Suspicion Score and KL-based methods, since it lacks directionality. Coordinated coalition behavior produces weak signals at the individual level. However, clustering for the suppression coalition and dimension-level entropy exceedance for the inflation coalition provide strong detection signals. This result is consistent with the Stackelberg model framework developed in Section 2.2, as coalition coordination leaves a trace detectable through community-level analysis.

### 5.6. Confidence Score System

A confidence score was calculated on the test data by combining the entropy deviation and KL divergence components for each dimension.

For each dimension q the confidence score combines the entropy-deviation and KL-divergence signals into a single integrity indicator in the [0, 1] interval through Equation (13):

(13) $$CS\left(q\right)=w_{H}\cdot exp(-\frac{\left|z_{\left\{\Delta H,q\right\}}\right|}{\lambda_{H}})+w_{KL}\cdot exp(-\frac{D_{KL,t}\left(q\right)}{\lambda_{KL}})$$

where $z_{\left\{\Delta H,q\right\}}$ is the standardized entropy deviation of dimension q, $D_{KL,t}(q)$ is its KL divergence from the reference, and $w_{H}+w_{KL}=1$. Each exponential term maps an undisturbed signal (zero deviation, zero divergence) to 1 and decays monotonically toward 0 as the distortion grows, so that values close to 1 indicate high data integrity and values close to 0 indicate severe corruption. The constants adopted in this study are $w_{H}=w_{KL}=0.50,\;\lambda_{H}=3,\;and\;\lambda_{KL}=0.25.$ The global confidence score is obtained as the geometric mean of the dimension-level scores, which prevents a single clean dimension from masking a corrupted one; on the test data, this yields a global CS of 0.70. The confidence score ranges between 0 and 1, where values close to 1 indicate high data integrity and values close to 0 indicate low integrity. Table 13 presents the confidence scores on a dimension basis.

The confidence score system constitutes a three-layered warning mechanism. The green level, corresponding to a CS greater than 0.75, indicates that data integrity is high and survey outputs can be used directly as inputs in decision processes. The yellow level, where CS is greater than 0.50 and less than or equal to 0.75, carries a medium level of risk. It is recommended that the purified results after preprocessing be presented alongside the raw results. The red level, where CS is less than or equal to 0.50, demonstrates that data integrity is severely threatened and additional verification mechanisms must be consulted before making decisions regarding the relevant dimension.

On the test data, the Management Leadership and Belonging dimension produced a red warning while the Research and Development dimension produced a yellow warning. These two dimensions are the ones directly targeted by the Coordinated Suppression and Coordinated Inflation groups, respectively. The fact that the system correctly flags the manipulated dimensions while keeping the three non-manipulated dimensions at the green level confirms that the confidence score mechanism exhibits a meaningful discrimination capacity in an institutional context.

### 5.7. Policy Impact Analysis

The confidence score system provides the opportunity to quantify the distinct impacts of raw and verified results on policy decisions. Table 14 compares the raw mean of each dimension with its verified mean, where coordinated and straight-lining manipulation groups are excluded.

The most critical finding revealed by the policy impact analysis is that manipulation produces deviations in different directions across distinct dimensions. Although the dimension ranking remains unchanged following verification, absolute scores and interdimensional distances differentiate significantly.

The most striking pattern is observed in the Management Leadership and Belonging dimension. This dimension possesses the second-lowest mean in the raw data, with a score of 3.374. However, the mean increases to 3.415, corresponding to a rise of 0.041 points, when the Coordinated Suppression group is excluded. This directionality implies that coordinated suppression portrays this dimension more negatively than its actual state. An institutional evaluation based on raw data would perceive the issue in the Management dimension as being larger than reality and allocate resources according to an incorrectly calibrated priority sequence. In contrast, within the Research and Development dimension, the raw mean is 0.145 points higher than the verified mean, due to the artificial elevation created by the Coordinated Inflation group. In this instance, the issue is depicted as being smaller than its true magnitude.

These two deviations in opposite directions concretely exemplify the fundamental claim of the study. Institutional prioritization based on unverified raw data can systematically mis-calibrate both the magnitude and the direction of problems. Because the direction of manipulation varies from dimension to dimension, a simple general correction such as decreasing all means by a fixed amount cannot eliminate this distortion. Therefore, dimension-selective detection is mandatory.

### 5.8. Comparative Evaluation

To evaluate the relative performance of the proposed framework, two standard anomaly-detection methods were applied comparatively on the test dataset defined in Section 5.1.

The Z-score method calculates the standard scores for the mean response values and the extremity ratios of the participants and flags those falling outside plus or minus two standard deviations as anomalies. Straight-lining detection was included in this method as an additional rule. Isolation Forest [43] is an ensemble tree method utilizing participant features including mean response standard deviation, extremity ratio, and completion time as inputs. The expected contamination rate was matched with the actual manipulation rate of the test data, which is 26.2 percent. Table 15 presents the comparative performance of the three approaches on the test data.

The results reveal that each method is strong in a different detection dimension. The Z-score method produces perfect Precision but misses the majority of manipulated participants, with a low Recall of 0.410. The reason for this is that the holistic means of dimension-selective coalition members overlap with those of honest participants. Isolation Forest establishes a balance between Precision and Recall but fails to reach a high performance level in any metric. Both standard methods are unable to produce the KL time-series pattern that indicates dimension-selective coalition behavior.

The strength of the proposed framework lies in its layered structure rather than a single metric. While straight-lining detection produces a perfect F1 value for Type I, Type II KL divergence monitoring detects coalition entry windows before data collection is completed. Furthermore, entropy band monitoring localizes both coalition targets at the dimension level, and clustering analysis reveals the suppression coalition with 76.5 percent purity, a determination which the Individual Suspicion Score fails to capture alone. No single standard method can perform these functions simultaneously. This comparison demonstrates that the contribution of the framework is not a new single detection mechanism, but an integrated architecture capturing distinct manipulation signatures across different layers.

At the participant level, the integrated framework attains an F1 of 0.709 (Precision 0.918, Recall 0.577), exceeding both the Z-score (F1 = 0.582) and Isolation Forest (F1 = 0.577) baselines. The recall ceiling reflects the dimension-selective coalition members and the noise-injection group, which are addressed at the population and dimension levels rather than through participant-level flags; an AUC is not reported for the integrated configuration, because it combines a deterministic filter with a clustering decision rather than a single continuous score.

## 6. Discussion

This section evaluates the empirical findings across four axes, including theoretical contributions, practical implications, methodological limitations, and comparative positioning with existing methods.

### 6.1. Theoretical Contributions

The primary theoretical contribution of this study is the formal characterization of strategic agent interactions in institutional survey systems through entropy dynamics. Proposition 2 and Proposition 3, which were theoretically expressed and empirically supported in previous sections, demonstrate that the trace left by manipulation in the data space is measurable in an information-theoretic sense. The entropy values of dimensions subjected to coordinated manipulation, specifically, Management Leadership and Belonging along with Research and Development, fell outside the reference control band, whereas non-manipulated dimensions remained within the band (Table 10), serving as the concrete empirical counterparts of these propositions. This finding offers an original contribution by merging strategy-based literature with information theory. The signal distortion theoretically predicted by Crawford and Sobel’s [19] cheap talk model is empirically observed in this study as entropy traces over institutional survey data. In this process, manipulative behavior is conceptualized not merely as a preference deviation but as a mechanism that transforms the information content of the response distribution.

Scenario analysis (Table 11; summarized in Table 12) reveals that distinct manipulation types leave distinguishable signatures on entropy dynamics. Type I and Type II manipulations create a radical homogenization that locks Shannon entropy into a single value, forming the most prominent trace in the data space. Coordinated suppression and coordinated inflation, which represent the sub-components of Type IV, produce a dimension-selective distortion where the entropy disruption in the target dimension appears alongside preserved normality in other dimensions. This selectivity pattern provides a unique signature that can be utilized to detect coalition behavior. Conversely, Type III noise injection produced the least distinctive signature, contrary to expectations. The fact that the AUC values for this type remained close to the random threshold of 0.50 in Monte Carlo experiments (Table 11) confirms that random response behavior statistically overlaps with honest participants who naturally exhibit high variance on the Likert scale. This overlap explains why noise injection is inherently a strategy that is difficult to detect.

Perhaps the strongest theoretical contribution of the study is the direct empirical evidence it generates regarding the necessity of a multi-layered design. In the test data (Section 5.4), the mean Individual Suspicion Scores of the coordinated coalition groups, which equal 0.388 for suppression and 0.346 for inflation, are not distinctively higher than the mean of the honest group, which stands at 0.417, and are actually slightly lower. This outcome appears paradoxical at first glance, but is theoretically predictable because coalition members exhibit deviations solely in a single target dimension while producing honest responses across all other dimensions, rendering their individual response profiles holistically indistinguishable from those of honest participants. Under these conditions, where an individual indicator remains insufficient, the integrated layers of the framework involving dimension-level entropy monitoring, KL divergence, and hierarchical clustering provide a systematic detection capacity through cumulative evidence accumulation. Indeed, the suppression coalition was captured in the clustering layer with 76.5% purity and ~59% recall, whereas the inflation coalition was identified via dimension-level entropy exceedance (Table 10; Section 5.4). This structure exhibits a conceptual overlap with the Dempster–Shafer theory of evidence combination formalized by Dempster [36], since the principle that weak individual signals can be amplified with support from independent evidence sources is well-documented in the literature on decision-making under uncertainty. This study uniquely adapts the relevant principle to the context of survey integrity.

### 6.2. Practical Implications

The KL divergence time-series findings (Figure 2) reveal that manipulation can be detected before the data collection process is finalized. The alarm signal generated by the coordinated suppression group became prominent during the final third of the data collection period, when this group concentrated its entries into the system. This early warning capacity grants institutional quality offices the opportunity for proactive intervention while the survey process is still ongoing. This overcomes a structural constraint of traditional survey quality assessment, as standard approaches can only evaluate response errors after data collection is fully completed, often well after decision-making processes have already moved forward.

Policy impact analysis results (Table 14) quantitatively demonstrate that the difference between raw and verified means can generate significant deviations in institutional prioritization decisions. What is particularly striking here is that manipulation produces deviations in opposite directions across distinct dimensions. In the Management Leadership and Belonging dimension targeted by coordinated suppression, the raw mean falls below the verified mean, meaning the problem in this dimension is portrayed as larger than it actually is. Conversely, in the Research and Development dimension targeted by coordinated inflation, the raw mean is 0.145 points higher than the verified mean (Table 14), depicting the issue as smaller than its true magnitude. This bidirectional distortion leads to an important practical conclusion, as a simple and general correction strategy, such as lowering all means by a fixed amount, cannot eliminate this deviation. Only a dimension-selective detection and correction mechanism can ensure that resource allocation is accurately calibrated. When resource allocation is conducted over unverified signals, institutional improvement programs risk being designed with excess capacity in certain areas and insufficient capacity in others.

To successfully implement the developed framework in institutional environments, fulfilling three primary conditions is crucial. First, to construct a healthy reference profile, historical survey data spanning at least two to three years must be digitally accessible; a five-year reference pool was utilized in this study. Second, it is mandatory for the survey system to record the start and end timestamps on a participant basis, as both the temporal anomaly component and the capacity of the KL time series to localize coalition windows depend on this data. Finally, the institutional quality office is expected to possess the capability to correctly interpret the risk of false positives. The developed system is a quantitative support tool presented to the decision-maker rather than a full mathematical automation. Observing this distinction is critically important in preventing the system from facing institutional legitimacy issues.

### 6.3. Methodological Limitations and Boundary Conditions

A boundary condition concerning the validation design must be stated explicitly. The Stage 2 and Stage 3 results in Section 5 were obtained on a test set whose honest participants were sampled from the same reference distributions that define the control limits, and whose manipulation groups were injected by construction. This shared generative origin means that the low false-positive rate reported for the honest majority is partly a property of the design rather than independent evidence of specificity, and that the detection rates for Types I, II, and IV reflect performance against manipulations whose statistical form is known a priori. The framework’s behavior on genuinely independent corruption—manipulation whose distributional form is not derived from the reference—therefore remains to be established. Two measures mitigate this concern. First, the reference profile itself rests on five years of real institutional data (Section 4), so the honest baseline is empirically grounded rather than fully synthetic. Second, a leave-one-year-out protocol, in which one historical year is withheld from reference construction and treated as an unlabeled monitoring period, would provide a partially external test; preliminary application of this protocol is left to future work. Readers should interpret the operating characteristics in Section 5.5 as internal-validity evidence under controlled conditions, to be complemented by external validation on independently collected manipulated data.

The empirical identification process revealed a distinct operational boundary regarding the clustering performance, specifically manifested as a residual gap comprising nine members of the suppression coalition who evaded both individual scoring mechanisms and hierarchical clustering clusters (Section 5.4). This sub-group reported target-dimension responses that closely mimicked the boundary parameters of the honest cohort, leveraging a subtle strategic shift rather than a blunt extreme score injection. Consequently, their statistical footprints remained embedded within the high-density regions of the reference distribution, demonstrating that highly sophisticated, low-intensity agent coordination can partially bypass entropy-boundary checks, and highlighting the need for integrating downstream behavioral features.

Type III noise injection constitutes the most prominent structural limitation of the framework (Table 11). Participants who respond randomly do not diverge from natural high-variance honest participants in terms of either the Individual Suspicion Score or KL-based indicators. This limitation stems from the epistemological nature of noise injection rather than the framework design because aimless randomness is qualitatively entirely different from a rule-based deviation and does not produce a measurable distance from the reference distribution since it lacks a statistical direction. One approach to address this issue involves integrating additional metadata layers related to response speed and session behavior into the analysis. Genuine noise injection is typically accompanied by very rapid responding and inconsistent interaction speeds across questions, and this information carries the potential to strengthen the temporal component of the framework.

The Individual Suspicion Score alone cannot produce sufficient group differentiation. This limitation is fuelled by two primary sources. First, the fact that coalition members provide strategic responses focused solely on specific dimensions complicates a holistic individual differentiation. Second, the weights of the Individual Suspicion Score components were not optimized over a labelled validation set, and the baseline values based on theoretical priority ranking, which assign 40% to extremity, 35% to the content component, and 25% to the temporal component, were maintained. It is anticipated that a weight optimization conducted with labelled manipulation data would increase individual detection performance.

The weak differentiation of the coordinated inflation group in the clustering analysis is another boundary condition that warrants attention. Because this group applies a soft inflation to its target dimension, its members overlap with honest participants who naturally assign high scores. The final detection of this group was achieved through dimension-level entropy exceedance instead of clustering. This situation should be interpreted not as a flaw but as a validation of the layered design of the framework, showing that distinct manipulation types are captured at different layers and no single layer is sufficient for all types.

The framework assumes that the reference data is largely free of manipulation. The absence of any straight-lining profiles during four of the reference years, spanning 2021 to 2024, supports this assumption. However, whether more subtle forms of strategic behavior existed in previous years cannot be directly known. In a scenario where the reference itself is systematically contaminated, the framework will take a distorted profile to be considered as a normal baseline, and the warning signal it generates for actual manipulation will weaken. Two complementary measures are proposed to mitigate this risk, which include updating the reference profile after pre-processing once each new period is completed, and monitoring the holistic entropy values of the reference years for interannual consistency to reduce the weight of outlier years.

Finally, the statistical power of the sliding window analysis directly depends on the number of participants per window. During periods when the participation rate remains low, the number of observations within the window increases the variance of the entropy estimation and elevates the risk of false positives. Under these conditions, reducing the window size or recalibrating the thresholds becomes necessary.

### 6.4. Comparative Positioning with Existing Methods

The proposed framework diverges from standard anomaly-detection methods across three primary dimensions. First, methods based on Z-scores and interquartile ranges measure the deviation of individual responses from the central tendency. On the test data (Table 15), the Z-score method produced perfect Precision at 1.000 but could only achieve a Recall of 0.410 because coalition members target only a single dimension while producing responses conforming to the reference distribution across other dimensions, and holistic statistics conceal this selective pattern.

Second, machine learning-based methods such as Isolation Forest and local outlier factor either require labelled training data or produce reliable results only within large sets of observations. On the test data, Isolation Forest balanced Precision and Recall, where both metrics equaled 0.577, but failed to achieve a high-performance level in any metric (Table 15). Within institutional environments characterized by an annual survey cycle, the labelled data and large sample conditions required by these methods cannot be structurally provided.

Third and most importantly, both standard methods are unable to produce the KL time-series pattern and the early warning capacity that signal dimension-selective coalition behavior. The proposed framework effectively fills these gaps. The model does not require labelled training data and can be directly implemented in any institution where historical survey data is available. The framework models both temporal and dimension-selective anomaly patterns across distinct layers and transforms its outputs into confidence scores and warning levels that the decision-maker can interpret. This final feature satisfies a requirement that is becoming increasingly critical for trustworthy artificial intelligence systems regarding explainability. The contribution of the framework is therefore not to propose a new single detection mechanism but to offer an integrated architecture that captures distinct manipulation signatures within complementary layers and converts them into institutional decision support.

## 7. Conclusions

Data uncertainty arising from strategic actor interactions in institutional survey systems emerges as a dynamic phenomenon at the intersection of information theory and decision-support systems. The entropy-based framework developed in this study empirically demonstrates that the traces left by manipulation in the data space can be quantified. The degree of uncertainty contained in information sources is transformed into a direct indicator of data reliability within the system. In contrast to the traditional survey quality literature that views participants as passive respondents, this approach positions them as strategic actors driven to shape institutional outcomes. Consequently, the signal distortion dynamics predicted in game theory are observed as concrete entropy traces over institutional survey data.

The answers provided by the study to the four research questions can be summarized as follows. Within the scope of the first research question, it is demonstrated that strategic manipulation can be statistically detected through temporal deviations in entropy and KL divergence where dimensions subjected to coordinated manipulation fall outside the reference control band while non-manipulated dimensions remain within the band. The Monte Carlo experiments conducted under the second research question reveal the statistical operating characteristics of the framework. Individual suppression and inflation manipulations are detected with almost perfect success across all intensity levels, whereas noise injection constitutes the structural boundary of the framework and coordinated coalition behavior produces weak signals at the individual level but strong signals at the community level. Under the third research question, it is documented that individual strategic response misreporting and coordinated coalition behavior leave distinguishable signatures on entropy dynamics, though the coalition signature becomes visible only through community-level analysis, including clustering and dimension-level entropy monitoring. Within the scope of the fourth research question, it is shown that detection outputs can be transformed into institutional decision support in the form of confidence scores and warning levels and that undetected manipulation produces quantitatively measurable deviations in policy decisions.

The research results indicate that collective manipulation movements must be monitored through community behavior patterns rather than individual suspicion indicators. This is perhaps the most significant methodological finding of the study, because the individual suspicion scores of coordinated coalition members are indistinguishable from those of honest participants, while the same members leave a strong and interpretable trace at the community level. The developed multi-layered analysis structure possesses the capacity to generate early warnings over time-series data before the survey process is fully completed. Furthermore, the findings quantitatively demonstrate that institutional prioritizations and resource allocations based on unverified raw data can produce systematic deviations. The fact that manipulation produces deviations in opposite directions across different dimensions indicates that this distortion cannot be resolved by a simple general correction and can only be accurately assessed through a dimension-selective detection mechanism. This situation elevates the data reliability problem from being a purely technical quality dimension to a direct institutional governance risk.

The limitations of the study also point to future research directions. Session metadata and click speeds can be included in the analysis to overcome the structural detection difficulty introduced by random responding or noise injection. Designing adaptive reference architectures that reflect institutional development instead of a fixed reference profile represents another important research area. Decentralized multi-agent learning approaches that share latent state information across distributed agents, as in Zhou et al. [41], carry the potential to increase statistical stability, particularly in organizations with low participation rates. Optimizing the individual suspicion score components over labelled validation data could enhance detection performance at the individual level. Furthermore, adapting this framework to different sectoral areas, such as patient satisfaction surveys in healthcare systems or public service evaluations, will pave the way for comparative methodological research.

The success of institutional decision processes is directly related to the integrity of the data feeding these processes. In environments where participants are motivated to influence institutional outcomes, absolute and unquestioning trust in data creates a serious management vulnerability. This study offers an integrated methodology that makes the strategic risks in question visible and manageable. The universal metrics of information theory demonstrate that the institutional governance approach must treat data not as a passive input but as a dynamic object that needs to be actively audited.

## Figures and Tables

**Table 1:** Contributions and limitations of the literature in the referenced fields.

Field	Key Contribution	Primary Limitation
Survey quality and DSS	Response error typology and measurement validity	No strategic actor model
Game theory and mechanism design	Theoretical foundation of strategic behavior	No empirical detection tool for survey systems
Data integrity and poisoning	Mathematical characterization of manipulation	No human agent or institutional context
Entropy-based decision-making	Information-theoretic quantification of uncertainty	No dynamic monitoring under strategic corruption
This study	Integrates four fields in a single framework	

**Table 2:** Summary table for the three-stage framework.

Stage	Input	Core Calculation	Output
Reference	Historical survey data from $t_{-n}$ to $t_{-1}$	$P_{ref}$, $\mu_{H}$, $\sigma_{H}$, UCL, LCL, $\Sigma_{ref}$	Baseline entropy profile
Monitoring	New period response stream	$H_{t}$, $D_{KL,t}$, $\Delta Corr_{t}$	Warning signals
Scoring	Stage 2 outputs and individual responses	$MAS_{t}$, $ISS_{i}$, and cluster analysis	Anomaly decisions

**Table 3:** Raw data summary by year.

Year	Raw Participants	Number of Observations	Raw Mean
2021	218	3488	3.249
2022	231	3696	3.365
2023	247	3952	3.412
2024	259	4144	3.445
2025	284	4544	3.642
Total	1239	19,824	3.434

Note. Raw Mean denotes the arithmetic mean of all Likert responses on the 1–5 scale, pooled over all 16 items and for all participants of the corresponding year, computed on the raw data before any preprocessing (i.e., before straight-lining removal and imputation).

**Table 4:** Straight-lining detection by year.

Year	Participants	Straight-Lining (All-5)	Straight-Lining (All-1)	Total Straight-Lining	Percentage
2021	218	0	0	0	0.0%
2022	231	0	0	0	0.0%
2023	247	0	0	0	0.0%
2024	259	0	0	0	0.0%
2025	284	5	1	6	2.1%

**Table 7:** Reference correlation matrix.

	Management Leadership	Education and Training	Research and Development	Social Contribution	Working Environment
Management Leadership	1	0.332	0.335	0.349	0.362
Education and Training	0.332	1	0.313	0.277	0.262
Research and Development	0.335	0.313	1	0.307	0.305
Social Contribution	0.349	0.277	0.307	1	0.259
Working Environment	0.362	0.262	0.305	0.259	1

**Table 8:** Stage 1 reference parameters.

Parameter	Value/Description	Parameter	Value/Description
Number of clean reference observations	19,728	Global $P_{ref}$ mean	3.461
Number of clean reference participants	1233	Number of bootstrap iterations	1000
Number of reference years	5, covering 2021 to 2025	Control limit method	±2 bootstrap κ with 95.4 percent
Weighting method	Time proximity, ranging from 0.10 to 0.30	$\Sigma_{ref}$ correlation range	0.259 to 0.362
Global $H_{ref}$	2.2166	2025 straight-lining rate	2.1 percent, equivalent to 6 out of 284
Normalized $H_{ref}$	0.9546	Global mean impact of straight-lining cleanup for 2025	−0.015 points

**Table 9:** Test dataset participant group design.

Group	N	Share	Manipulation Type	Target Dimension
Honest	220	73.8%	None close to reference	Not applicable
Straight-lining, All 5	28	9.4%	Type II Individual inflation	All dimensions
Straight-lining, All 1	4	1.3%	Type I Individual suppression	All dimensions
Coordinated Suppression	22	7.4%	Type IV Coalition suppression	Management Leadership and Belonging
Noise Injection	14	4.7%	Type III Random response	All dimensions
Coordinated Inflation	10	3.4%	Type IV Coalition inflation	Research and Development
Total	298	100%		

**Table 10:** Comparison of dimension entropy with reference control limits.

Dimension	$H_{ref}$	LCL	UCL	$H_{test}$	Status
Management Leadership and Belonging	2.2206	2.2049	2.2364	2.2562	UCL Exceeded
Education and Training	2.1526	2.1334	2.1718	2.1665	Normal
Research and Development	2.1779	2.1572	2.1986	2.2106	UCL Exceeded
Social Contribution	2.1275	2.0948	2.1603	2.1422	Normal
Working Environment	2.2456	2.2300	2.2612	2.2387	Normal

**Table 11:** Monte Carlo experiment results.

Type	Intensity	Precision	Recall	F1	AUC
Type I Suppression	5%	1.000	1.000	1.000	1.000
Type I Suppression	10%	1.000	1.000	1.000	1.000
Type I Suppression	15%	0.975	1.000	0.987	1.000
Type I Suppression	20%	1.000	1.000	1.000	1.000
Type I Suppression	30%	1.000	1.000	1.000	1.000
Type II Inflation	5%	1.000	1.000	1.000	1.000
Type II Inflation	10%	1.000	1.000	1.000	1.000
Type II Inflation	15%	0.980	1.000	0.990	1.000
Type II Inflation	20%	1.000	1.000	1.000	1.000
Type II Inflation	30%	1.000	1.000	1.000	1.000
Type III Noise	5%	0.460	0.290	0.356	0.493
Type III Noise	10%	0.162	0.091	0.113	0.526
Type III Noise	15%	0.179	0.133	0.150	0.523
Type III Noise	20%	0.173	0.111	0.134	0.490
Type III Noise	30%	0.327	0.263	0.289	0.518
Type IV Coalition	5%	0.132	0.071	0.085	0.656
Type IV Coalition	10%	0.189	0.104	0.129	0.633
Type IV Coalition	15%	0.243	0.162	0.191	0.617
Type IV Coalition	20%	0.299	0.229	0.257	0.610
Type IV Coalition	30%	0.423	0.296	0.340	0.616

**Table 12:** Manipulation types: detection traces and performance comparison.

Type	Manipulation	Entropy Effect	Straight Lining	Clustering	Overall Difficulty
I	Individual Suppression All 1	H ↓↓ distinct	Full detection	Direct	Low
II	Individual Inflation All 5	H ↓↓ distinct	Full detection	Direct	Low
III	Noise Injection	H ↑ slight	Undetectable	Weak	High
IV-A	Coalition Suppression	H ↑ UCL exceedance	Undetectable	Strong 76.5 percent	Medium
IV-B	Coalition Inflation	H ↑ UCL exceedance	Undetectable	Weak	Medium to High

Note. In the Entropy Effect column, ↓↓ denotes a pronounced decrease in Shannon entropy relative to the reference band, and ↑ denotes an increase; “UCL exceedance” indicates that the increase is large enough for the observed entropy to exceed the upper control limit of the reference band.

**Table 13:** Confidence score table, by dimension.

Dimension	H Deviation	KL	CS	Warning Level
Management Leadership and Belonging	+4.52 σ UCL exceedance	0.084	0.47	Red
Education and Training	+1.44 σ Normal	0.005	0.80	Green
Research and Development	+3.17 σ UCL exceedance	0.018	0.64	Yellow
Social Contribution	+0.90 σ Normal	0.022	0.83	Green
Working Environment	−0.89 σ Normal	0.011	0.85	Green
Global			0.70	Yellow

**Table 14:** Policy impact matrix.

Dimension	Raw Mean	Verified Mean	Difference
Social Contribution	3.743	3.639	−0.104
Education and Training	3.647	3.515	−0.132
Research and Development	3.577	3.432	−0.145
Management Leadership and Belonging	3.374	3.415	+0.041
Working Environment	3.345	3.226	−0.119

**Table 15:** Comparative performance evaluation.

Method	Precision	Recall	F1	AUC	Primary Limitation
Z-score	1.000	0.410	0.582	0.803	Low Recall
Isolation Forest	0.577	0.577	0.577	0.783	Balanced but weak
Proposed Straight-lining	1.000	1.000	1.000	-	Only Type I and II
Proposed Integrated	0.918	0.577	0.709	-	Layered architecture

## Data Availability

The raw data supporting the results of this article may be requested from the authors with written permission from the relevant institution.

## Appendix A. Proofs of Propositions

This appendix provides the proofs of Propositions 1–3, whose formal statements are given in Section 2.2 (Proposition 1) and Section 2.3 (Propositions 2 and 3). The notation follows the multi-agent formalization $S=\left(I,Q,A,\Omega ,U,R\right)$ introduced in Section 2.1 and the entropy and Kullback–Leibler definitions of Equations (1) and (2).

### Appendix A.1. Proof of Proposition 1

This subsection proves Proposition 1 as stated in Section 2.2. The game-theoretic setting is recalled below for completeness.

The leader commits to a monitoring mechanism that induces a detection probability $\pi \left(\delta \right)\in \left[0,1\right]$, non-decreasing and differentiable in the manipulation intensity δ≥0, with $\pi \left(0\right)=\pi_{0}$. The follower chooses δ≥0 to maximise the expected utility

$$U\left(\delta \right)=\left(1-\pi \left(\delta \right)\right)\;G\left(\delta \right)-\pi \left(\delta \right)\;K=G\left(\delta \right)-\pi \left(\delta \right)\;\left(G\left(\delta \right)+K\right),$$

where G is the gain from undetected manipulation, with $G\left(0\right)=0$, $G^{\prime }\left(\delta \right)>0$, and $G^{\prime \prime }\left(\delta \right)\leq 0$, and K>0 is the penalty incurred upon detection.

**Claim.** If the marginal deterrence dominates the marginal gain,

$$\pi^{\prime }(\delta )\;\left(G\left(\delta \right)+K\right)\geq \left(1-\pi \left(\delta \right)\right)\;G^{\prime }(\delta )\;\;\mathrm{for}\;\mathrm{all}\;\delta \geq 0,(*)$$

and in particular if $\pi^{\prime }\left(0\right)\geq G^{\prime }\left(0\right)/K$, then the unique best response is $\delta^{*}=0$: honest reporting is dominant and the equilibrium manipulation intensity collapses to zero.

**Proof** **of** **Proposition** **1.** Differentiating the utility,

$$U^{\prime }\left(\delta \right)=\left(1-\pi \left(\delta \right)\right)\;G^{\prime }\left(\delta \right)-\pi^{\prime }\left(\delta \right)\;\left(G\left(\delta \right)+K\right).$$

Under condition $\left(*\right)$, $U^{\prime }\left(\delta \right)\leq 0$ for all δ≥0, so U is non-increasing on [0,∞) and attains its maximum at $\delta^{*}=0$, with $U\left(0\right)=0$. Any δ>0 then gives $U\left(\delta \right)\leq 0=U\left(0\right)$, so deviating from honesty is weakly dominated; under strict inequality in $\left(*\right)$ it is strictly dominated, and $\delta^{*}=0$ is the unique best response. Evaluating $\left(*\right)$ at δ=0, and using $G\left(0\right)=0$ and $\pi \left(0\right)=\pi_{0}<1$, gives the sufficient condition

$$\pi^{\prime }\left(0\right)\;K\geq \left(1-\pi_{0}\right)\;G^{\prime }\left(0\right)\;\Leftrightarrow \;\pi^{\prime }\left(0\right)\geq \frac{\left(1-\pi_{0}\right)\;G^{\prime }\left(0\right)}{K},$$

which reduces to $\pi^{\prime }\left(0\right)\geq G\prime \left(0\right)/K$ in the worst case $\pi_{0}\to 0$. Because the leader can choose a mechanism whose detection sensitivity $\pi^{\prime }\left(0\right)$ is as large as the entropy- and KL-based monitor permits, any scheme satisfying this bound deters all manipulation: the equilibrium intensity is $\delta^{*}=0$ and honest responding is the dominant strategy. This establishes the deterrence result referenced in Section 2.2. □

### Appendix A.2. Proof of Proposition 2

This subsection proves Proposition 2 as stated in Section 2.3. The setting and notation are recalled below for completeness.

Fix a target item q with reference distribution $P_{ref}=\left(p_{1},\ldots ,p_{k}\right)$ over the Likert categories A={1,…,k}, with $p_{a}>0$ for all a. Honest respondents draw from $P_{ref}$. Let a coalition consisting of c manipulating agents among the n respondents of the current survey period—that is, a coalition of relative size ρ=c/n∈(0,1]—draw instead from a manipulation distribution $P_{man}\neq P_{ref}$, with mass shifted toward the low categories under suppression (Type I, Type IV-A) and toward the high categories under inflation (Type II, Type IV-B). Writing $m_{a}=P_{man}\left(a\right)$, the observed period distribution is the mixture

$$P_{t}\left(a\right)=\left(1-\rho \right)\;p_{a}+\rho \;m_{a}.$$

**Claim.** $D_{KL}\left(P_{ref}\;∥\;P_{t}\right)>0$ for every ρ∈(0,1], and on a neighbourhood of ρ=0 the divergence is strictly increasing and convex with leading term

$$D_{KL}\left(P_{ref}\;∥\;P_{t}\right)=\frac{\rho^{2}}{2ln2}\sum_{a\in A}\frac{{\left(p_{a}-m_{a}\right)}^{2}}{p_{a}}+o\left(\rho^{2}\right),$$

expressed in bits.

**Proof** **of** **Proposition** **2.** Since $P_{man}\neq P_{ref}$, there is at least one category with $m_{a}\neq p_{a}$; hence, $P_{t}\neq P_{ref}$ whenever ρ>0. By Gibbs’ inequality $D_{KL}\left(P_{ref}\;∥\;P_{t}\right)\geq 0$, with equality if and only if $P_{t}=P_{ref}$; therefore, $D_{KL}\left(P_{ref}\;∥\;P_{t}\right)>0$ for all ρ>0. Define

$$g\left(\rho \right)=\sum_{a\in A}p_{a}{log}_{2}\frac{p_{a}}{P_{t}\left(a\right)},\;\;P_{t}\left(a\right)=p_{a}+\rho \;\left(m_{a}-p_{a}\right),$$

so that $g\left(0\right)=0$. Differentiating with respect to ρ,

$$g^{\prime }\left(\rho \right)=-\frac{1}{ln2}\sum_{a\in A}\frac{p_{a}\left(m_{a}-p_{a}\right)}{P_{t}\left(a\right)},\;\;g^{\prime }\left(0\right)=-\frac{1}{ln2}\sum_{a\in A}\left(m_{a}-p_{a}\right)=0,$$

because both $P_{ref}$ and $P_{man}$ sum to one. The second derivative is

$$g^{\prime \prime }\left(\rho \right)=\frac{1}{ln2}\sum_{a\in A}\frac{p_{a}{\left(m_{a}-p_{a}\right)}^{2}}{P_{t}{\left(a\right)}^{2}}\geq 0,\;g^{\prime \prime }\left(0\right)=\frac{1}{ln2}\sum_{a\in A}\frac{{\left(m_{a}-p_{a}\right)}^{2}}{p_{a}}=\frac{1}{ln2}\;\chi^{2}\left(P_{man},P_{ref}\right)>0.$$

The last equality is definitional. The Pearson chi-square divergence of $P_{man}$ from $P_{ref}$ is defined as

$$\chi^{2}\left(P_{man},P_{ref}\right)\;=\;\sum_{a\in A}\frac{{\left(m_{a}-p_{a}\right)}^{2}}{p_{a}}.$$

Evaluating $g^{\prime \prime }$ at ρ=0 gives $P_{t}\left(a\right)=p_{a}$, so each summand $p_{a}\;{\left(m_{a}-p_{a}\right)}^{2}/P_{t}{\left(a\right)}^{2}$ reduces to $p_{a}\;{\left(m_{a}-p_{a}\right)}^{2}/p_{a}^{2}={\left(m_{a}-p_{a}\right)}^{2}/p_{a}$, and summing over a∈A yields exactly $\chi^{2}\left(P_{man},P_{ref}\right)$. Since $P_{man}\neq P_{ref}$ and $p_{a}>0$ for all a, at least one summand is strictly positive, whence $g^{\prime \prime }\left(0\right)>0$. The appearance of the chi-square divergence here is natural: it is the local second-order approximation of the Kullback–Leibler divergence, consistent with the Taylor expansion stated in the claim. We now show in detail that g is strictly increasing on $\left(0,1\right)$, and that this holds uniformly over all admissible pairs $\left(P_{ref},P_{man}\right)$. Since $p_{a}>0$ for all a∈A, for every ρ∈[0,1) we have

$$P_{t}\left(a\right)=\left(1-\rho \right)\;p_{a}+\rho \;m_{a}\;\geq \;\left(1-\rho \right)\;p_{a}>0,$$

so g is twice continuously differentiable on [0,1). Because $P_{man}\neq P_{ref}$, there exists at least one category $a^{*}$ with $m_{a^{*}}\neq p_{a^{*}}$; its contribution $p_{a^{*}}\;{\left(m_{a^{*}}-p_{a^{*}}\right)}^{2}/P_{t}{\left(a^{*}\right)}^{2}$ to $g^{\prime \prime }\left(\rho \right)$ is strictly positive for every ρ∈[0,1). Hence, $g^{\prime \prime }\left(\rho \right)>0$ on the whole interval [0,1), not merely at ρ=0. By the fundamental theorem of calculus and $g^{\prime }\left(0\right)=0$,

$$g^{\prime }\left(\rho \right)=g^{\prime }\left(0\right)+{\int_{0}^{\rho }g}^{\prime \prime }\left(s\right)\;ds\;=\;{\int_{0}^{\rho }g}^{\prime \prime }\left(s\right)\;ds>0\;\;\mathrm{for}\;\mathrm{all}\;\rho \in \left(0,1\right).$$

Therefore, g is strictly convex and strictly increasing on $\left(0,1\right)$, with a strict minimum of 0 at ρ=0. This argument uses only the two assumptions $p_{a}>0$ for all a and $P_{man}\neq P_{ref}$; it does not depend on the particular shapes of $P_{ref}$ and $P_{man}$, which is why the conclusion is uniform over all admissible pairs. At the boundary point ρ=1, two cases must be distinguished. If $m_{a}>0$ for every a with $p_{a}>0$, then $P_{t}\left(a\right)>0$ also at ρ=1, and the argument extends to the closed interval $\left[0,1\right]$ by continuity. If, instead, $m_{a}=0$ for some a with $p_{a}>0$ (for example, a degenerate straight-lining coalition concentrating all mass on a single category), then $P_{t}\left(a\right)\to 0$ as $\rho \to 1^{-}$ and $g\left(\rho \right)\to +\infty$; the divergence remains strictly increasing along the approach to the boundary, and the statement of the proposition is understood on [0,1) in this degenerate case. The second-order Taylor expansion of g about ρ=0 yields the stated leading term. Any coordinated deviation (ρ>0, $P_{man}\neq P_{ref}$ ) therefore produces a strictly positive divergence of order $\rho^{2}$, which exceeds the control threshold β once ρ or $∥P_{man}-P_{ref}∥$ is sufficiently large. □

### Appendix A.3. Proof of Proposition 3

This subsection proves Proposition 3 as stated in Section 2.3.

Under the no-manipulation assumption (honest and interannually stationary responses), the $N_{t}$ observations of item q in reference year t are independent and identically distributed, drawn from $P_{ref}$. Let ${\hat{P}}_{t}$ denote the empirical distribution and

$${\hat{H}}_{t}=-\sum_{a\in A}{\hat{P}}_{t}\left(a\right){log}_{2}{\hat{P}}_{t}\left(a\right)$$

its plug-in entropy.

**Claim.** ${\hat{H}}_{t}$ concentrates around $H\left(P_{ref}\right)$ and lies within

$$\left[{LCL}_{q},\;{UCL}_{q}\right]=H_{ref}\pm \kappa \;\sigma_{H}$$

with probability approximately $2\Phi \left(\kappa \right)-1$, where Φ denotes the cumulative distribution function of the standard normal distribution $N\left(0,1\right)$; this probability equals about 0.954 for κ=2. A value outside this band signals a violation of the honesty or stationarity assumption.

**Proof** **of** **Proposition** **3.** Let $\hat{N}=\left({\hat{N}}_{1},\ldots ,{\hat{N}}_{k}\right)$ denote the multinomial counts of the $N_{t}$ observations across the k. Likert categories, and let ${\hat{P}}_{t}=\hat{N}/N_{t}$ be the empirical distribution. By the multivariate central limit theorem for the multinomial distribution,

$$\sqrt{N_{t}}\;\left({\hat{P}}_{t}-P_{ref}\right)\overset{d}{\to }N_{k}\left(0,\Sigma_{P}\right),\;\;\Sigma_{P}=diag\left(P_{ref}\right)-P_{ref}\;P_{ref}^{T}.$$

The entropy functional $H\left(p\right)=-\sum_{a\in A}p_{a}{log}_{2}p_{a}$ is continuously differentiable at every interior point of the probability simplex (i.e., wherever $p_{a}>0$ for all a), with gradient components

$$\frac{\partial H}{\partial p_{a}}=-\left({log}_{2}p_{a}+\frac{1}{ln2}\right).$$

Applying the first-order delta method to the smooth map $p\mapsto H\left(p\right)$ at $p=P_{ref}$ yields

$$\sqrt{N_{t}}\;\left({\hat{H}}_{t}-H\left(P_{ref}\right)\right)\overset{d}{\to }N\left(0,\sigma_{\infty }^{2}\right),\;\;\sigma_{\infty }^{2}=\nabla H{\left(P_{ref}\right)}^{T}\;\Sigma_{P}\;\nabla H\left(P_{ref}\right).$$

Because every row of $\Sigma_{P}$ sums to zero, adding the constant 1/ln2 to all coordinates of the gradient leaves the quadratic form unchanged; hence only the $-{log}_{2}p_{a}$ part of the gradient contributes, and the variance evaluates in closed form as

$$\sigma_{\infty }^{2}=\sum_{a\in A}p_{a}{\left({log}_{2}p_{a}\right)}^{2}-(\sum_{a\in A}p_{a}{log}_{2}p_{a}{)}^{2}=\sum_{a\in A}p_{a}{\left({log}_{2}p_{a}\right)}^{2}-H{\left(P_{ref}\right)}^{2}.$$

Hence ${\hat{H}}_{t}$ is asymptotically Gaussian about $H\left(P_{ref}\right)$ with standard deviation $\sigma_{H}\approx \sigma_{\infty }/\sqrt{N_{t}}$. The bootstrap procedure of Section 3.1 and Section 4.4 resamples the reference responses and recomputes the time-weighted entropy, yielding a consistent estimate ${\hat{\sigma }}_{boot}$ of $\sigma_{H}$; the band $H_{ref}\pm \kappa \;{\hat{\sigma }}_{boot}$ therefore covers ${\hat{H}}_{t}$, with probability approaching $2\Phi \left(\kappa \right)-1$. Conversely, suppose the period distribution is manipulated, $P_{t}=\left(1-\rho \right)P_{ref}+\rho P_{man}$ with ρ>0. By the strict concavity of the entropy functional, $E\left[{\hat{H}}_{t}\right]\to H\left(P_{t}\right)\neq H\left(P_{ref}\right)$; for ρ large enough, this mean shift exceeds $\kappa \sigma_{H}$ and pushes ${\hat{H}}_{t}$ outside the band, with probability tending to one. Manipulation-free entropies thus remain within the reference band, while manipulated ones depart from it. □
